# Effects of Climate Change on Plant Population Growth Rate and Community Composition Change

**DOI:** 10.1371/journal.pone.0126228

**Published:** 2015-06-03

**Authors:** Xiao-Yu Chang, Bao-Ming Chen, Gang Liu, Ting Zhou, Xiao-Rong Jia, Shao-Lin Peng

**Affiliations:** State Key Laboratory of Biocontrol, School of Life Sciences, Sun Yat-Sen University, Guangzhou, 510006, China; Chinese Academy of Forestry, CHINA

## Abstract

The impacts of climate change on forest community composition are still not well known. Although directional trends in climate change and community composition change were reported in recent years, further quantitative analyses are urgently needed. Previous studies focused on measuring population growth rates in a single time period, neglecting the development of the populations. Here we aimed to compose a method for calculating the community composition change, and to testify the impacts of climate change on community composition change within a relatively short period (several decades) based on long-term monitoring data from two plots—Dinghushan Biosphere Reserve, China (DBR) and Barro Colorado Island, Panama (BCI)—that are located in tropical and subtropical regions. We proposed a relatively more concise index, Slnλ, which refers to an overall population growth rate based on the dominant species in a community. The results indicated that the population growth rate of a majority of populations has decreased over the past few decades. This decrease was mainly caused by population development. The increasing temperature had a positive effect on population growth rates and community change rates. Our results promote understanding and explaining variations in population growth rates and community composition rates, and are helpful to predict population dynamics and population responses to climate change.

## Introduction

It has been widely predicted that global climate change will affect biodiversity [1–3], species distribution and population growth [4–6]. Forests, as indispensable resources on Earth, have tremendous ecological, economic, social, and aesthetic value. Tree species distribution and forest composition will undergo substantial shifts in the future [7–11]. Changes in forest community composition may exert impacts on net primary productivity and carbon storage [12, 13]. It is essential to determine how climate factors influence forest population growth and community composition [14].

Average population growth rates are usually expected to be stable, but patterns of variation in population growth rates are different for different species. Variation in population growth rates is related to species birth, growth, reproduction and death [8, 15–17]. A recent study considered forest population dynamics, particularly the rate at which forest distributions might shift under climate change [6]. Change in a forest community is related to species-specific responses of the tree species in that community to climate change. In terms of population dynamics, dominant species respond differently to changing environmental conditions [18, 19], thereby giving rise to a change in community composition. For instance, Feeley et al. (2007) [20] found a widespread decrease in tree growth rates for species occurring in large forested dynamic plots at Barro Colorado Island, Panama (BCI), and the changes in growth were significantly associated with regional climate changes. Not long after, the authors revealed that, in this same plot, there have been consistent, directional changes in the tree species composition towards a significantly greater representation of species with greater drought tolerance, which was consistent with long-term climate change [21]. Declines of population growth rates were found in other studies, and these declines were usually climate-correlated [22]. Furthermore, traditional analyses usually assume that tree species composition or population growth rate is constant and fail to capture the temporal dynamics of these factors, making it difficult to attribute the change to environmental conditions [20, 22]. Thus, quantifying the temporal growth dynamics of population in the context of community change when the influences of climate variations are of interest is necessary.

Tropical and subtropical forests are major biodiversity hotspots harbouring many species and are recognised as important global carbon sinks and sources [18, 19, 23–25]. Long-term directional changes in the community composition of tropical forests have been reported recently, and these changes have been attributed to climate change [21, 26]. Studies have analysed the effects of climate change on the composition of various plant communities in many areas, including deserts [27], grasslands [28]and lowland forests [29]. Actually, climate-driven impacts on long-term community changes are difficult to disentangle from changes driven by stand development [30]. Although, the causes underlying these changes, specifically the contribution of long-term climate change vs. successional recovery from past disturbances, remain debated [21]. To increase the accuracy and reliability of the assessment, the community development process should be considered when one examines the effects of climate change on community composition change.

To understand the effects of temporal environmental changes on community composition, it is important to identify the rates at which species have already responded to recent climate change [31]. Studies have indicated that climate change influences community composition [32, 33], but few quantified the influence of climate change on community composition change. To better understand the dynamics of community composition in a changing world, it is necessary to quantify community composition change rate.

Herein, we aimed first to compose a method for calculating the community composition change rate and to examine the influence of climate factors on community composition change using two long-term forest datasets (the observation data from DBR and BCI). Second, we aimed to conduct a linear model to separate the impacts of climate factors and time factors on species-specific population growth rate and to testify the impacts of relatively faster climate change on community composition change rate within a relatively short period (several decades).

## Methods

### Database

We employed two long-term datasets for use in our analysis. One is a dataset containing 30 years of data from DBR (Dinghushan Biosphere Reserve, Guangdong province, China); the other contains approximately 25 years of data from BCI (Barro Colorado Island, Panama). DBR has a subtropical climate, and BCI has a tropical climate. Rare species were not included in this study, because they are more sensitive to disturbance and the variations of their numbers are more stochastic.

The DBR (23°10′N, 112°35′E) has a monsoon climate and is located in a subtropical, moist forest life zone. The annual mean temperature is 22.3°C and the annual mean rainfall is 1678 mm with a distinct seasonal pattern. Monthly average air temperature and precipitation, representing DBR's climatic regime, were obtained from the Gaoyao weather station [34]. The reserve is dominated by regional climax, evergreen broadleaf forest represented by the *Cryptocarya* community, which is dominated by *Cryptocarya chinensis*, *Cryptocarya concinna* and *Castanopsis chinensis*. Abundance of the 3 dominant species in two communities was investigated 9 times from 1955 to 1985. One community was *Cryptocarya* community, which was at the relatively stable stage; the other community was *Pinus-Castanopsis-Schima* community, which was at a relatively early stage of succession. There were very few human activities in the study area until 1980s and there was no big pest outbreak before 1985[35]. More than twelve 10×10 m^2^ quadrates were investigated in each of the communities. The average abundance (number of individuals) of 3 species in 2 communities for every investigation was recorded [36] (S1 Table). The climate data for monthly temperature and precipitation in DBR were provided by Guoyi Zhou (2012, pers. comm., 5 September).

The BCI plot (9°9'N, 79°51'W) covers an area of 50 ha; it was the first plot established by the CTFS (Center for Tropical Forest Science) and is undoubtedly one of the best-studied forests in the world [21]. The island receives an average of 2634 mm of rain per year, with a pronounced four-month dry season from mid-December through mid-April [37]. Daytime temperatures reach an average of 32°C, with night-time lows of approximately 23°C. This island has 1500 ha of diverse, moist, lowland, tropical forest, and has been protected from human disturbance for 70 years [38]. Abundance data collected from 1981 to 2005 on the 50 ha plot were obtained from the Center for Tropical Forest Science (http://www.ctfs.si.edu/) and monthly climate data were provided by Steven Paton (2012, pers. comm., 23 October). There were 319 species recorded for woody stems ≥ 10 mm diameter at breast height. Among these species, only species with an average of at least 10 individuals per ha for 6 censuses were included in our analysis because large percentage changes in abundance of rare species could be caused by minor, chance events [39]. Consequently, we finalised a list of 77 species for analysis (S2 Table). The total abundance of 77 species accounted for 91 percent of the total individuals on the plot.

### Calculation of population growth rate

To quantify population growth, we calculated relative population growth rate as:

$$\text{ln}\;\lambda =\text{ln}(N_{2}/N_{1})/(t_{2}-t_{1})$$ (1)

Where *t*_*2*_ *-t*_*1*_ is the time interval between two investigations; N_1_ and N_2_ are the numbers of individuals for a species in year t_1_ and t_2_. This is a common equation to calculate population growth rate [40, 41] and this model structure has been used to calculate the change of cover [27, 42]. We obtained 8 sequential intervals in DBR and 5 sequential intervals in BCI to estimate the species-specific population growth rate. Once the population growth rates were estimated, we determined the direction and rates of change in the parameters by calculating the linear least-squares regression coefficient (β) of lnλ vs. time separately for each of the species. For BCI, we also analysed the distribution of β to see the overall trend of population growth. We also analysed the relationship between the correlation coefficient and the abundance of each species to explore whether there is any influence of abundance on population growth rate.

### Calculation of community composition change rate

Based on the population growth rate, lnλ, of each species in a community, we composed a method to calculate the community composition change rate. The expression is as follows:

$$S\;\text{ln}\;\lambda =\sum_{i=1}^{N}\left|\text{ln}\;\lambda_{i}\right|/S$$ (2)

Here, Slnλ is the community composition change rate, expressed as the average of changes in population growth rate for the dominant species in a community, *S* is the number of species counted in the analysis and lnλ_i_ is the population growth rate of the *ith* species. The index Slnλ is a combination of the changes in biodiversity and population dynamics and reflects the rate of community change over a relatively short period. A larger Slnλ value indicates a greater change in community composition.

### Climatic factors in population growth and community composition change

Two basic models (the unrestricted exponential growth model and the restricted logistic growth model) are often used to simulate continuous population growth. Almost all populations undergo three phases: the increasing phase, the stabilising phase and the decreasing phase, but neither of the two models is able to describe the population declining stage. The interaction of three fundamental processes—(i) competition for resources, (ii) the effect of body size on resource use and (iii) the effect of plant density on growth and mortality—will affect the development of plant populations [43]. Given enough time, every population will decline. The limitation may come from the external environment or the self-limiting of a species. Although the logistic growth model has frequently been used in forest ecology, species replacement during forest development makes the population growth of the majority of species more likely to resemble a Gaussian curve (Eq 3).

$$f(t)=Ae^{-\frac{{\left(t-\mu \right)}^{2}}{2\sigma^{2}}}$$ (3)

In this equation, *μ* is the time when a species reaches its maximum abundance, *σ* is the time distance between *μ* and its maximum growth rate, and *A* is an adjusting parameter. We adopted both a logistic model and a Gaussian model to fit population growth for single species in each plot. We used the Akaike’s Information Criterion corrected for small sample size (AICc) to compare the fit results of the two models. Finally, we chose the Gaussian model as the population growth model for our analysis because the Gaussian model was a better fit than the logistic model for most species (3:1) (S1 and S2 Figs, S3 Table). We adopted the Gaussian model (Eq 3) to the model of population growth rate (Eq 1) and obtained the following model:

$$\text{ln}\;\lambda =\frac{\mu }{\sigma^{2}}-\frac{1}{2\sigma^{2}}(t_{1}+t_{2})$$ (4)

If σ^2^ = -1 / (2B) and μ = -A/ (2B), we acquire a simple model structure of population growth rate:

$$\text{ln}\;\lambda =A+B(t_{1}+t_{2})$$ (5)

Here, *A* and *B* are aggregate variables and theoretically can be determined by any factor that affect population growth. The linearity of this model structure makes the modelling process and the interpretation of each parameter more simple and accessible.

The annual mean temperature and precipitation adopted in our analysis are the most common and important climatic factors. It is very difficult to tell how climatic factors influence *A* and *B* with current knowledge. Thus, we constructed several possible models and compared their simulation results. We used two indices, Adjusted R-Squared and Akaike’s Information Criterion corrected for small sample size (AICc), to compare the fit results for six models. We planned to use the simplest model with the best fit for exploring the effects of climate on population growth (S4–S6 Tables). Finally, we chose the following model to test the influence of temperature and precipitation on population growth:

$$\text{ln}\;\lambda ∼T+P+(t_{1}+t_{2})$$ (6)

Here T is the annual average temperature and P is the annual precipitation. The time factor here *t*_*1*_ *+t*_*2*_ was derived by introducing Gaussian curve (Eq 3) to a common population growth rate model (Eq 1) in Ecology. A bigger value of the index can imply a bigger time interval, a more recent observation along a time sequence, or both. When one of them is fixed, the index can be interpreted by the other time factor.

If species-specific population growth is influenced by climatic factors, the community composition will be indirectly affected as well. To examine how climate influences community composition, we used linear regression analysis between the community composition change rate, Slnλ, and 6 different climatic factors (Tavg-annual mean temperature, Tmax-maximum temperature, Tmin-minimum temperature, Pavg-annual precipitation, Pmax-maximum precipitation and Pmin-minimum precipitation).

## Results

### Trends in air temperature and precipitation

**Fig 1:**
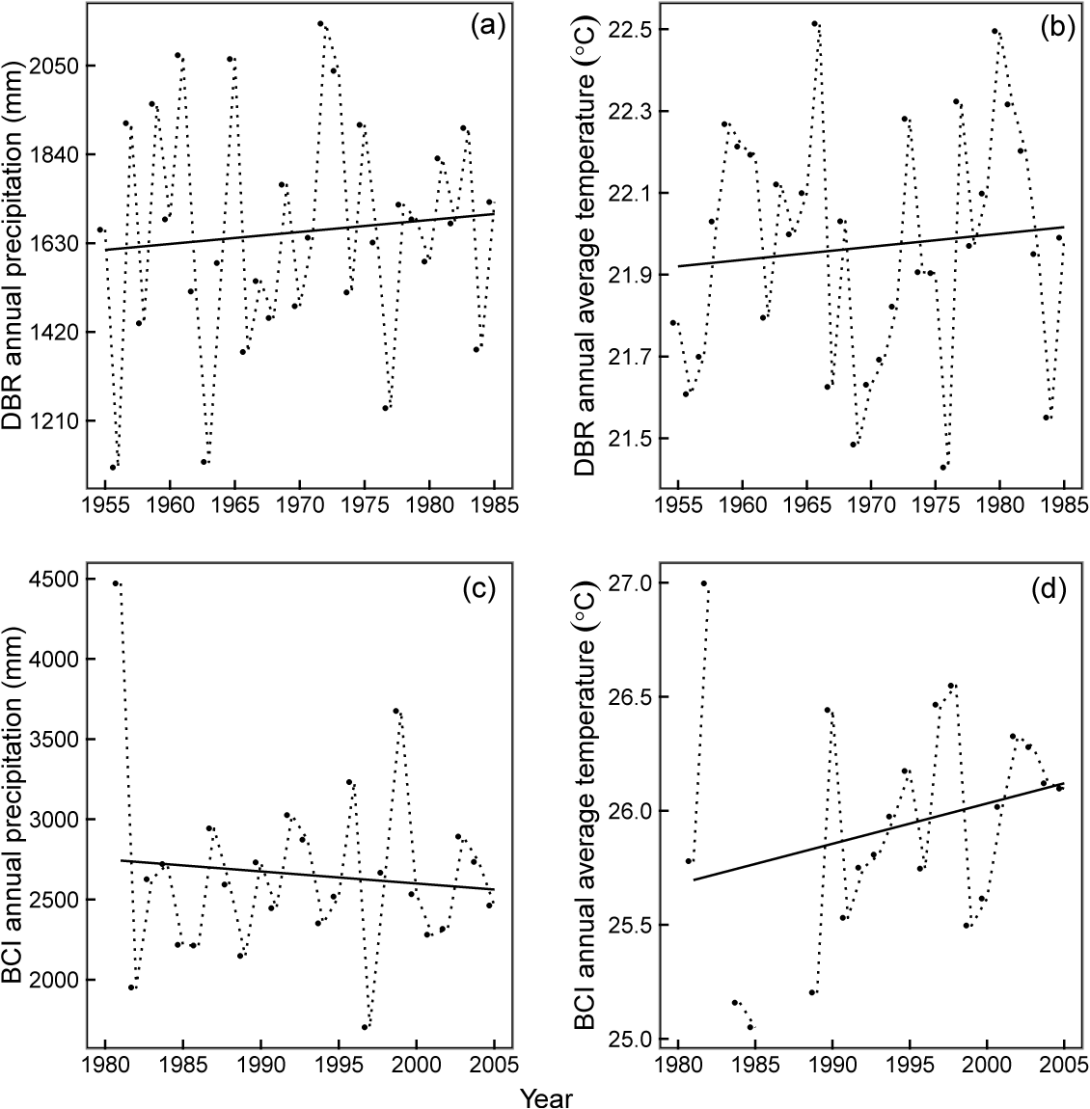
Trends in climate change (annual precipitation and annual mean temperature) in DBR and BCI. Temperature data were unavailable for a few months in the years 1983, 1986, 1987 and 1988 for BCI. There are 5 intervals for community I of DBR (1955–1963, 1963–1967, 1967–1978, 1978–1982, and 1982–1985), two intervals for community II of DBR (1978–1982 and 1982–1985) and 5 intervals for BCI (1981–1985, 1985–1990, 1990–1995, 1995–2000, and 2000–2005).

In DBR, the annual average temperature and the precipitation increased during the observation period (1955–1985) (Fig 1A and 1B), while in BCI, they showed different trends over time. In BCI, the annual average temperature increased while the annual average precipitation decreased from 1981 to 2005 (Fig 1C and 1D). The rate of increasing temperature in BCI was greater than that in DBR. When we compared the trend of annual average temperature at the two sites across a similar period (1981–2005), we found that the annual average temperature of both DBR and BCI showed a similar upward trend.

### Population growth rate

**Fig 2:**
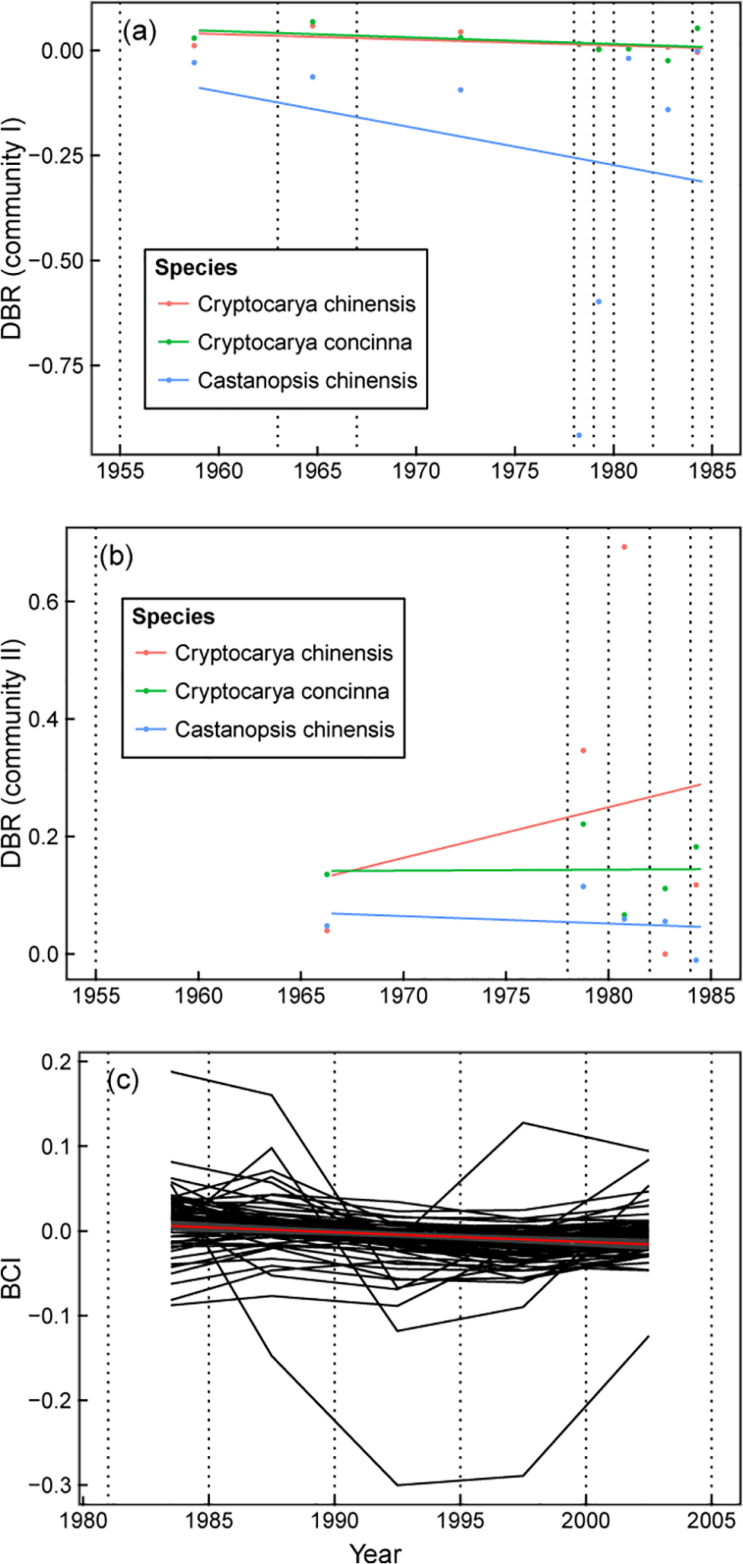
Changes in population growth rate, lnλ, over time in DBR and BCI. Vertical lines indicate census years, and points indicate the population growth rate, lnλ, per interval. Community I: *Cryptocarya* community; Community II: *Pinus-Castanopsis-Schima* community.

**Fig 3:**
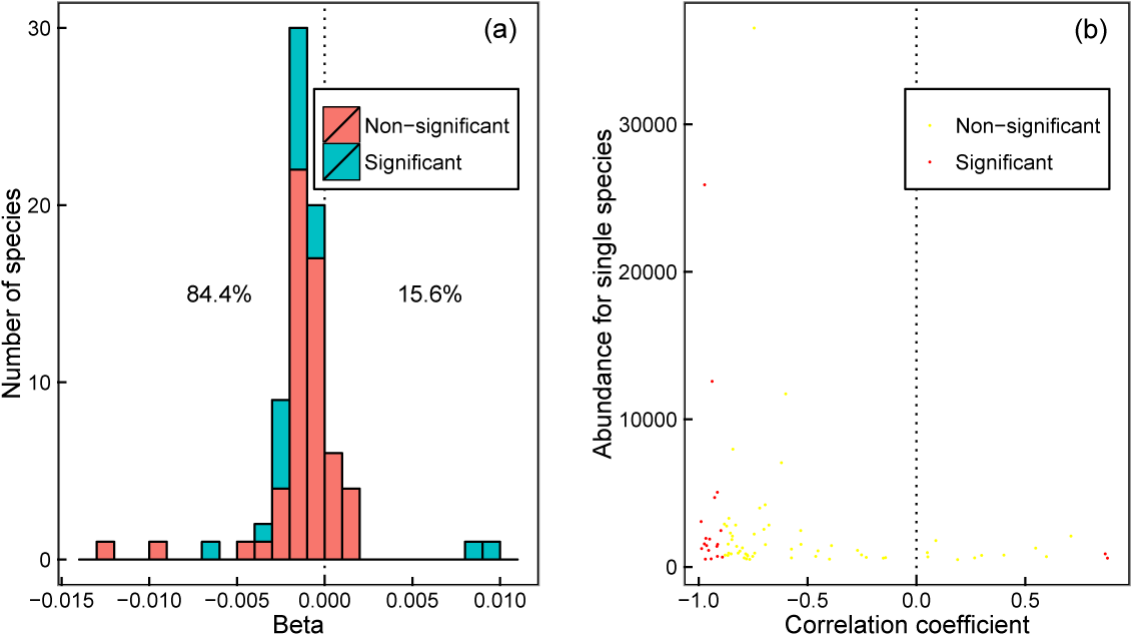
Distribution of the rate of change in population growth rate for 77 dominant species in BCI. (a) Distribution of the liner regression coefficient β of lnλ over time. (b) Relationship between abundance and the correlation coefficient for lnλ and time.

Population growth rates declined over time for most species in both DBR and BCI (Fig 2). In DBR, except for *C*. *chinensis* and *C*. *concinna* in community II (the *Pinus-Castanopsis-Schima* community), the population growth rates of the other species in the two communities decreased over time (Fig 2A and 2B). We analysed the distribution of the change rates for population growth rate in BCI, and the results showed that the population growth rates for most species (84.4%; 65 of 77 species) decreased (Figs 2C and 3A), with 27.7% of those species showing a significant decrease. Based on the 77 species in BCI, we carried out a correlation analysis between the population growth rate (lnλ) and time (the median of each five-year interval) to explore the relationship between the correlation coefficients and the abundances of species. The results indicated that many species with higher abundance showed decreasing trends of population growth rate (Fig 3B).

**Fig 4:**
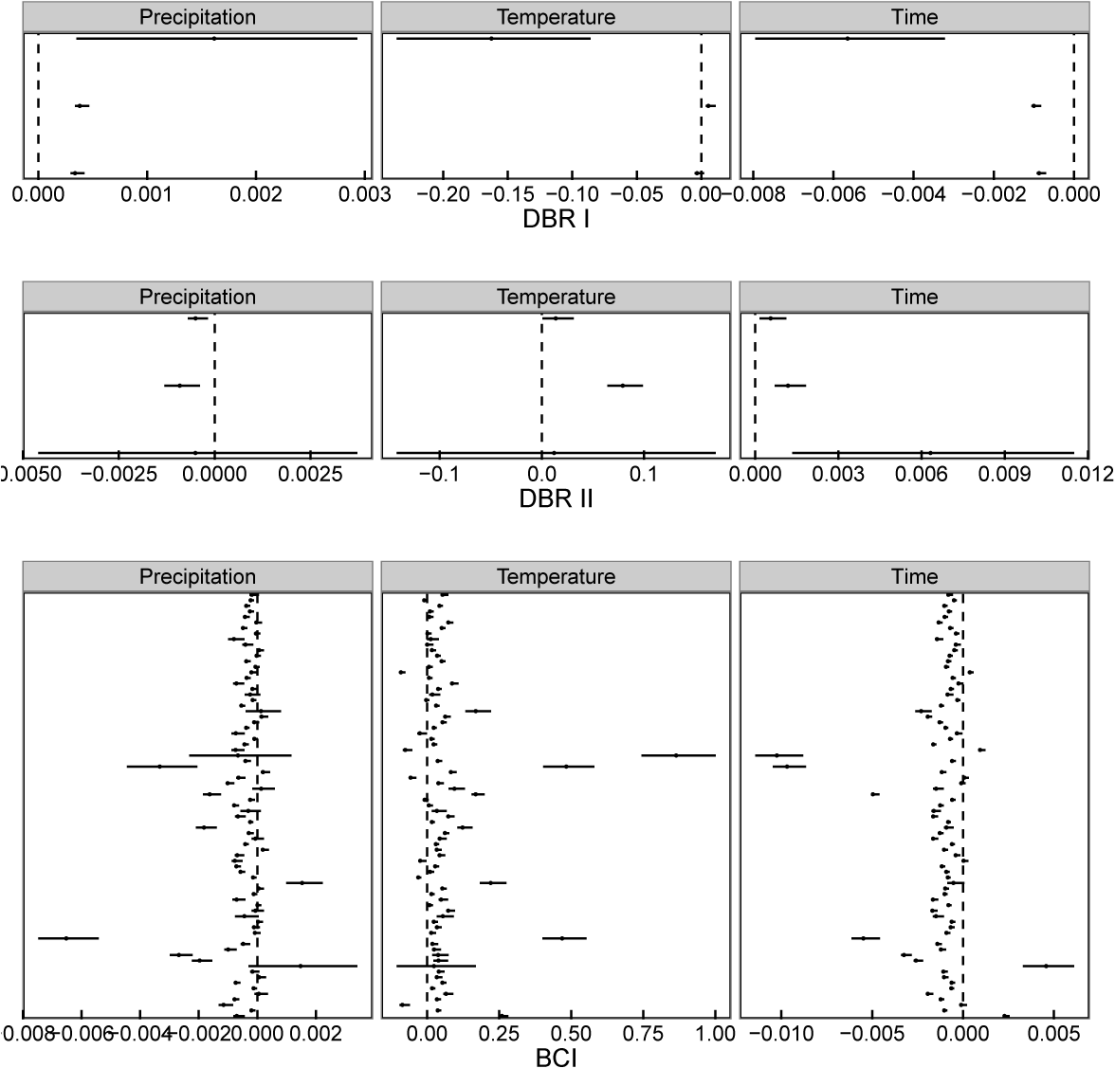
A multivariate linear regression revealed the effects of climate and time factors on population growth rate in DBR and BCI. The climate factors are annual mean precipitation (mm) and annual mean temperature (°C) for each time interval. The time factor is *t*_*1*_ *+t*_*2*_ (year), which is the sum of two observation years. All 3 models in community I, the model of *C*. *chinensis* in community II of DBR, and the 77 models in BCI are significant (P<0.05). Estimates and 95% confidence intervals of coefficients for three variables are indicated.

Species in the two communities on DBR responded differently to the time factor (*t*_*1*_ *+t*_*2*_). Population growth rate of the 3 species showed a negative response to time in community I, while showing a positive response in community II (Fig 4). The annual precipitation had a positive effect on community I but had a negative effect on community II. The annual average temperature had a positive effect on *C*. *concinna* in community I and on the 3 species in community II. For the 77 species in BCI, the population growth rate, lnλ, showed a negative response to the time factor (*t*_*1*_ *+t*_*2*_) (87%, significant), a negative association with the annual average precipitation (62.3%, significant) and a positive association with the annual average temperature (85.7%, significant).

### Community composition change rate

**Fig 5:**
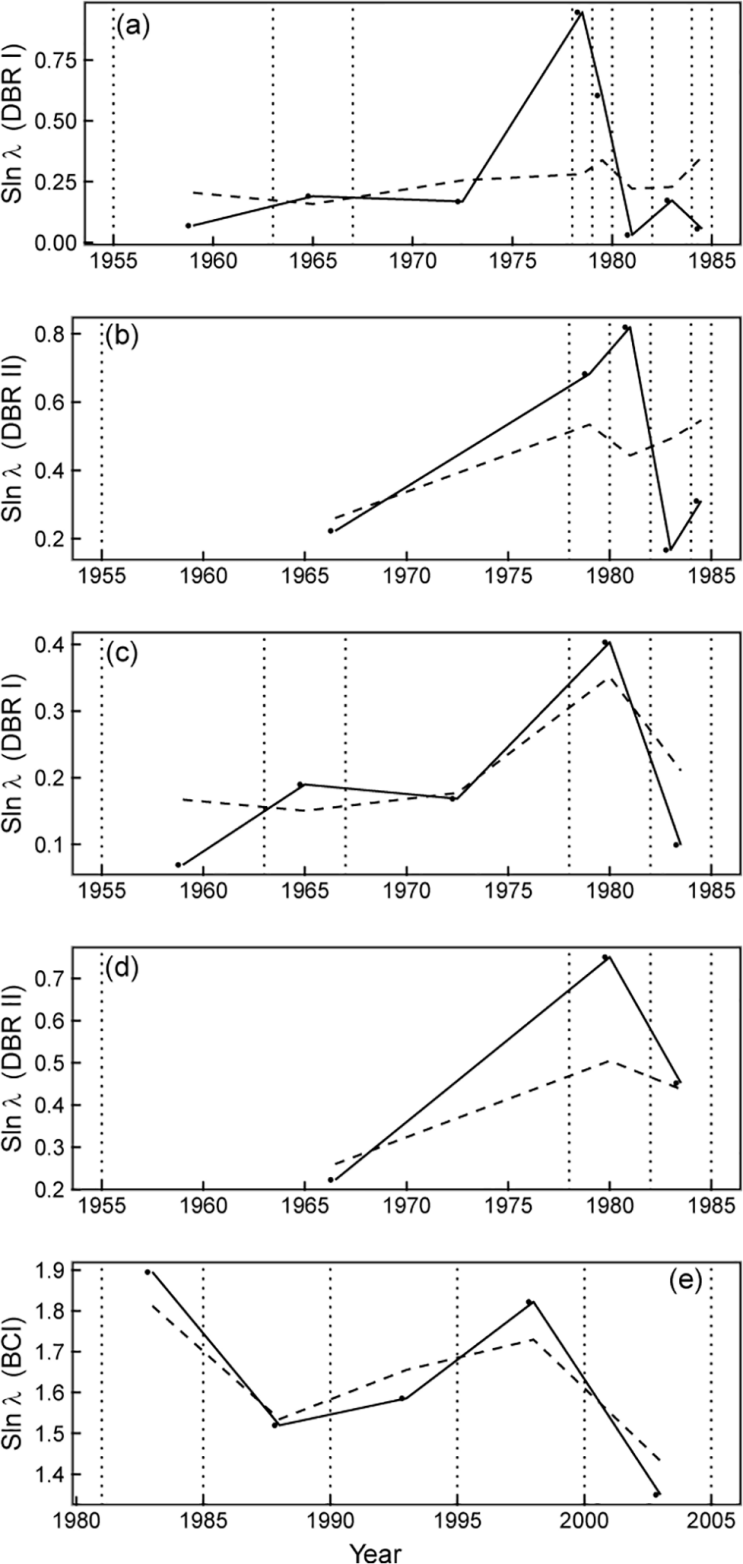
Variations of community composition change rate, Slnλ, during the study periods for DBR and BCI. The solid lines are based on the true values and the dashed lines are based on the fitted values. (a) Community I in DBR, (b) Community II in DBR, (c) Community I in DBR with adjusted intervals, (d) Community II in DBR with adjusted intervals, and (e) BCI.

We calculated the community composition change rate, Slnλ, based on the census data and the fitted data, separately. The fitted data were estimated based on the Eq 3. At first we calculated Slnλs for every adjacent census. The results showed that the fitted value and the true value displayed approximately the same variations over time in BCI (Fig 5E), while the variations of the fitted value and the true value for the two communities in DBR appeared to be different (Fig 5A and 5B). We noticed that the census intervals of DBR were very different, from 1 to 11 years for community I and 1 to 23 years in community II. So we combined some short intervals and recalculated the values and found that the fitted value and the true value displayed approximately the same variations (Fig 5C and 5D). The results suggest that when the community composition change rate is used as an index to compare different communities or different census times for one community, the time intervals should be identical.

**Fig 6:**
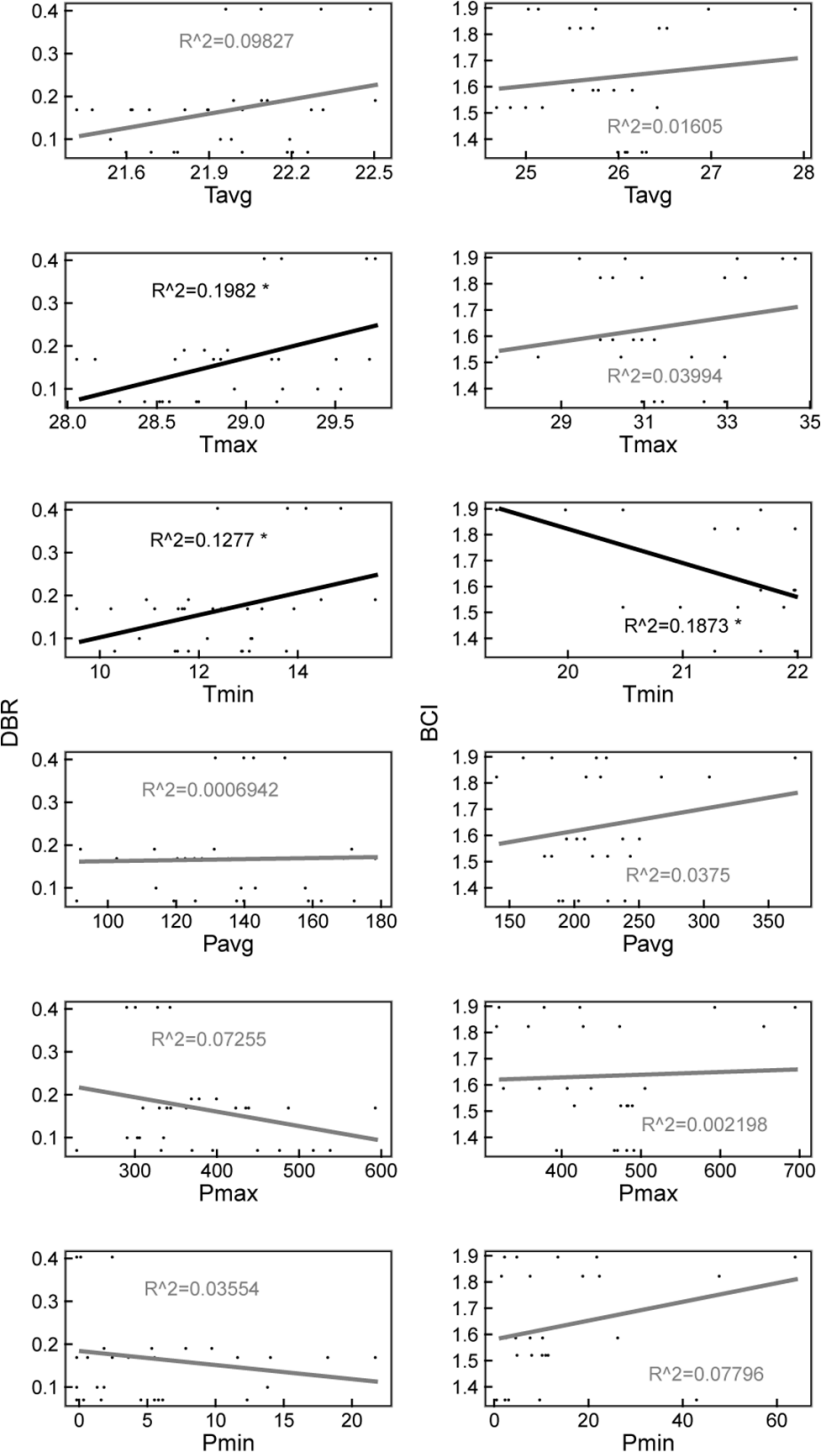
Effects of climate factors on community composition change rate, Slnλ, for DBR and BCI. Climate factors are the annual mean, maximum and minimum temperature (T_*avg*_, T_*max*_ and T_*min*_) and the annual mean, maximum, and minimum precipitation (P_*avg*_, P_*max*_ and P_*min*_). They were calculated based on monthly climate data. *P<0.05.

The linear regression analysis between climate factors and community composition change rate, Slnλ, showed that climate factors had no significant effects on Slnλ except on the maximum and minimum temperature in DBR and the minimum temperature in BCI. The maximum temperature had a positive effect on Slnλ, while the minimum temperature had a positive effect in DBR but a negative effect in BCI. The overall trend seems to link increasing temperature with increasing community composition change rate (5 in 6 regressions) (Fig 6).

## Discussion

### Impacts of climate change on population growth rate

**Fig 7:**
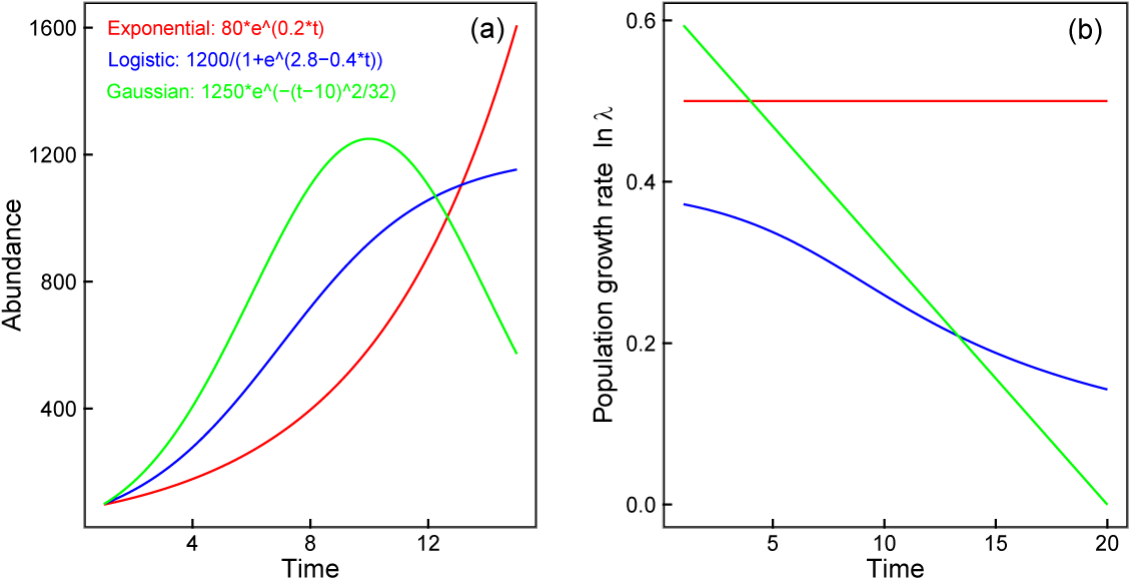
Models describing population growth and population growth rate. Changes in abundance and population growth rate, lnλ, over time simulated by the exponential growth model, logistic growth model, and Gaussian curve.

Population growth rate is the unifying variable linking the various facets of population ecology. It is the summary parameter of trends in population density or abundance [40]. For 3 species in DBR and 77 species in BCI, the population growth rate, lnλ, is negatively correlated with time for most species. This is consistent with previous studies [15, 21, 22, 44]. However, these studies could not determine the underlying causes of a declining population growth rate over time [15], or attributed this decline to environmental factors such as rainfall patterns [22] and survival variation [15]. The present study, however, indicates that this decreasing trend of population growth rate may be mainly a consequence of community development. When we use the exponential growth model, logistic growth model and Gaussian curve to simulate population growth, the population growth rate, lnλ, decreased over time except in the exponential growth model, where lnλ is constant (Fig 7). In addition, our multiple regression analysis showed that lnλ was negatively correlated with time for the majority of species (Fig 4). It can be concluded that for most species in a community, a decreasing population growth rate is inevitable. This is because the plant abundance and species richness will be close to the maximum environment carrying capacity with increasing population density and plant size. In the present study, community I in DBR was a mature forest; community II was in an earlier stage of succession than community I. The BCI forest was mature and relatively stable throughout the study period [21]. In addition to species-specific properties, the stage of population development is important in determining how the population growth rate of one species will respond to climatic factors.

However, previous studies usually measured the population growth rate by focusing on one time period and neglected to study the development of a population. Such neglect may affect predicted results because population growth rates change, and usually decrease, over time. Recently, using a specific database extracted from collected literature, a synthesis study examined how population growth rates varied within and among 50 plant species [15]. They found that population growth rates could vary greatly from species to species even within the same genus. However, population growth rates of different populations within the same species were similar, particularly if geographic distances among the populations were small and environmental factors were similar. These results can be explained by the present study, which demonstrates that the population growth rate of one species is not constant and is likely determined by the stage of community development and population growth. Different species usually appear at different stages of community development and have their own individual population growth parameters, even if they belong to the same genus [15]. Consequently, population growth rates vary greatly when species from different communities of different development stages are compared. Alternatively, nearby populations of the same species always belong to similar communities of nearly the same development stage. Therefore, populations with similar population growth rates may be geographically closer to each other.

Variations in environmental factors have substantial effects on plant population dynamics and are considered essential for realistic models of population dynamics [45, 46]. Temporal variation in environmental factors is important to population dynamics in terms of both long-term trends and short-term fluctuations [47]. Experimental warming has consistently produced an increase in vascular plant growth across the Arctic [48]. A recent study assessed the climate drivers of recent unfavourable silversword population dynamics and found that the declining population growth rates of silversword were associated with changing climatic conditions. Annual population growth rates were strongly tied to rainfall patterns [22]. A modelling study of a steppe plant community from eastern Idaho, USA, found strong effects of climate on the population growth in 2 of 3 target species. A 1% decrease in the previous year’s precipitation would lead to a 0.6% decrease in population growth for one species, and a 1% increase in summer temperature would result in a 1.3% increase in population growth for the other species [49].

Similarly, our study shows that an increasing annual mean temperature would increase population growth rates for the majority of species. However, a decreasing annual precipitation would also increase population growth rate in both DBR and BCI. This conflicts with other studies. One explanation is the persistent directional change in the flora toward an increased abundance of drought-tolerant species, possibly due to increasing temperature and/or a past decrease in rainfall, at least in BCI [21]. Another possible explanation is that during succession, as shade intolerant tree species gradually decline, the community tends to approach a climax that is dominated by mesophytic tree species [50], which might be favoured by less precipitation.

### Impacts of climate change on community composition change rate

Altered population growth of dominant species could have profound effects on community structure and ecosystem function [49]. Most previous studies on succession rate only considered the species composition change of a community [51–53], but did not take population dynamics into account. In fact, community change not only indicates the species composition change but also implies a proportional change of each species relative to one another [54]. One could not say a community is unchanged when no old species disappears and no new species established over years because the community composition often changes with different proportions of each species. Therefore, using the index Slnλ is more precise for detecting the rate of community change. Slnλ is determined by population growth rates of dominant species of a community, which can be predicted by environmental and temporal factors. Thus, community composition change rate, Slnλ, in a relatively short time period is predictable.

The community composition change rate, Slnλ, is determined by population growth rates of all species in a community. Accordingly, it can be inferred that at the stages when vast species replacement takes place, Slnλ should be very high. This new index can be very useful when we want to compare the stability of different communities during a time period or different stages of community changing process.

We used linear regression analysis to detect the relationship between climate and community change rate, Slnλ (Fig 6). Because species respond to climate individually, it is very difficult to predict the collective response. Our results suggest that a positive relationship exists between increasing temperature and increasing community change rate. Although decreasing precipitation has a positive effect on population growth rate for most populations, the overall effect on the community composition change rate seems complicated in our study. As we know, changes in precipitation can affect community composition by altering germination, seedling establishment, growth, and survival [55, 56]. In some studies, changes in the timing of rainfall have a greater influence on community composition than the amount of rainfall [57]. Another experimental study found that drought and warming treatments slowed down secondary succession in a Mediterranean climate, and indicated that future drier and warmer conditions may severely affect plant succession due to the existence of both abundance-dependent and species-specific responses [58]. For the BCI plot in our analysis, the impact of El Niño event in early 1983 caused immediate increases in tree mortality, and since then there has been a persistent directional change in the flora toward and increased abundance of drought-tolerant species[21]. These changes in composition were reflected by the community composition change rate Slnλ, which was extremely high in the 1980~1985 interval (Fig 5E).

From a long-term perspective, range shifts due to global warming will alter the structure and composition of communities and change the function of ecosystems [59]. As different species respond to climate change in various ways, species previously regarded as aliens will begin to appear in new communities. Species that can quickly adapt to new environmental conditions will outpace those that cannot [60]. Studies in Siberia revealed that a warmer climate will likely convert deciduous larch to evergreen conifer forests [61, 62]. When species diversity and abundance increase, community composition change rate generally will be accelerated. However, in a relatively short time period of 20 to 30 decades, species composition will not change significantly. Although climate changes influence population growth of each species, these changes will not fundamentally affect community change rate within a few decades.

To detect the community composition change rate over decades, we propose a relatively more concise index, Slnλ, which refers to an overall population growth rate based on the dominant species in a community. The variation of Slnλ over time is mainly determined by community development. Climate factors can influence the population growth rate and accordingly affect the community composition change rate. The present study indicates that increasing temperature has a positive effect on population growth rate and community change rate. Decreasing precipitation has a positive effect on population growth, but shows a complex effect on community change. As global warming will bring more precipitation to high latitudes in both winter and summer and less precipitation to low latitudes [63], further study is needed to reveal the relationships between the historical variations in climate and population dynamics in broader areas.

## Supporting Information

S1 Fig Change in abundance for 3 species from 2 plots in DBR. (DOC)

S2 Fig Change in abundance for 77 species from BCI. (DOC)

S1 Table Abundance (number/m^2^) of three dominant tree species from 1955 to 1985 in two communities for DBR, China. (DOC)

S2 Table List of 77 dominant species and their abundances from the BCI (Barro Colorado Island, Panama) tropical forest. (DOC)

S3 Table Comparisons of Logistic model and Gaussian model depending on the small-sample-size corrected version of Akaike information criterion (AICc). (DOC)

S4 Table Significance, Adjusted R-squared (Adj-R^2^), and the small-sample-size corrected version of Akaike information criterion (AICc) of 6 Models for 3 species from 2 plots in DBR. (DOC)

S5 Table Significance, Adjusted R-squared (Adj-R^2^), and the small-sample-size corrected version of Akaike information criterion (AICc) of 6 Models for 77 species in BCI. (DOC)

S6 Table Significance of coefficients, Adjusted R-squared (Adj-R^2^), and the Akaike information criterion (AIC) of 6 Models fitted with all species from each of the 3 plots (DBR, DBR and BCI), separately. (DOC)
