# Supplementary figures and images for: Effects of Climate Change on Plant Population Growth Rate and Community Composition Change
Source: PLoS One. 2015 Jun 3;10(6):e0126228. doi: 10.1371/journal.pone.0126228 (PMC4454569; doi:10.1371/journal.pone.0126228)

**S1 Fig.** **Change in abundance for 3 species from 2 plots in DBR.**


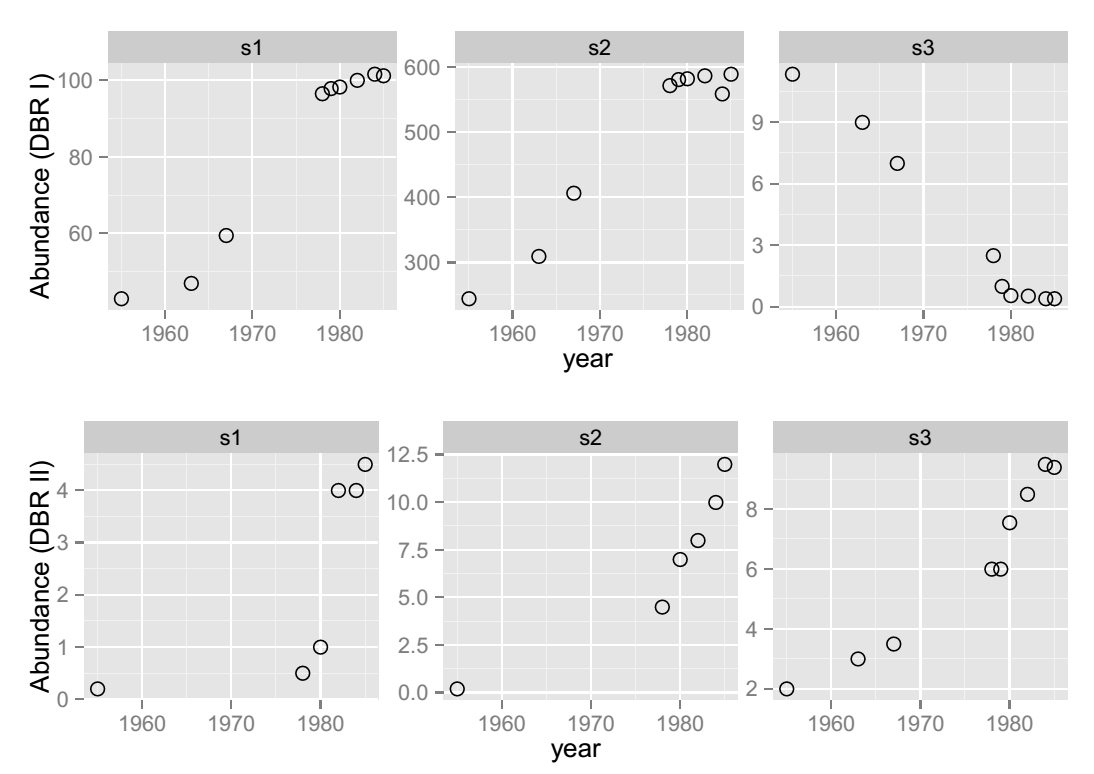

Supplement: S1 Fig — (DOC) [file pone.0126228.s001.doc]

**S2 Fig. Change in abundance for 77 species from BCI.**


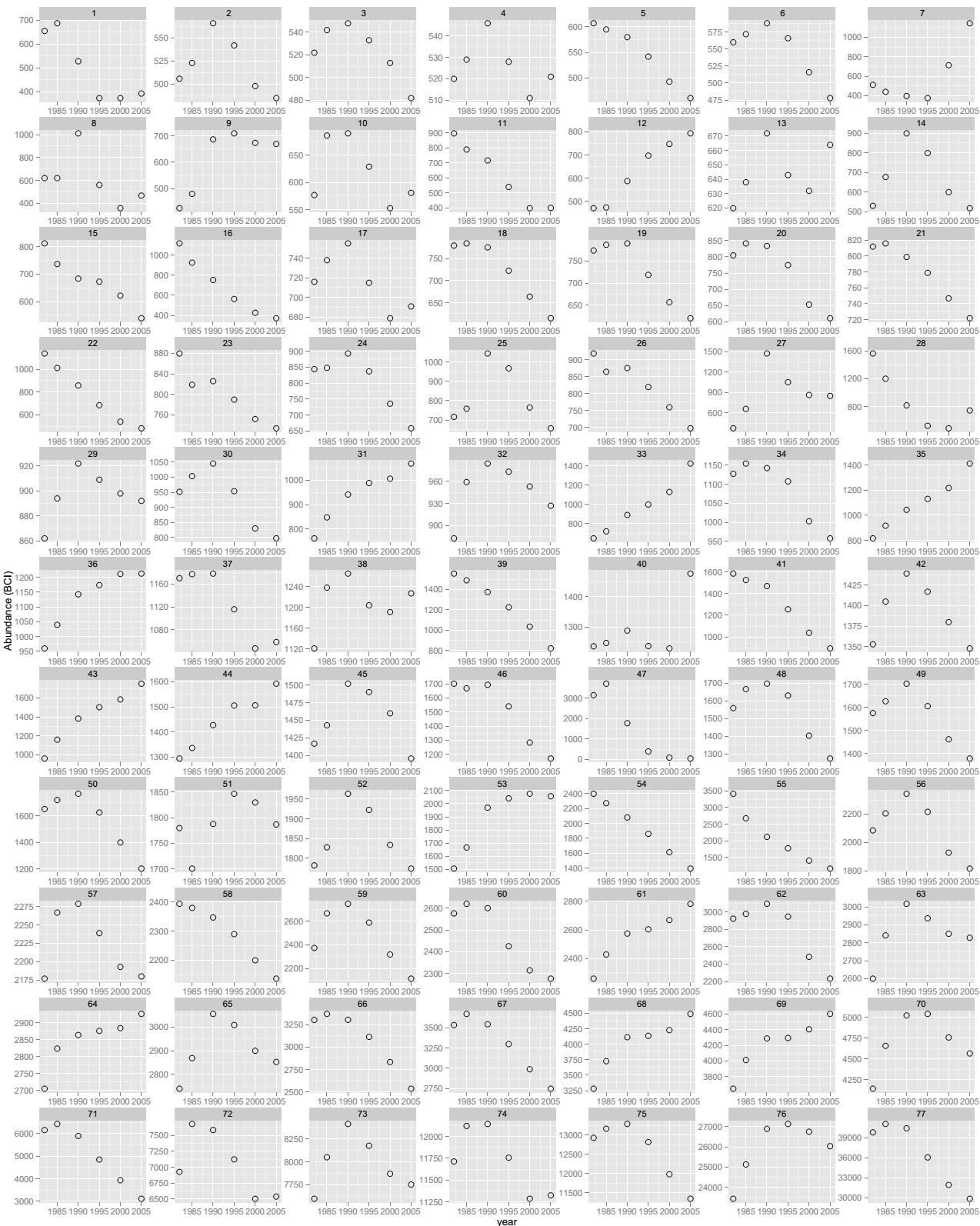

Supplement: S2 Fig — (DOC) [file pone.0126228.s002.doc]
