# Supplementary material for: Effects of Climate Change on Plant Population Growth Rate and Community Composition Change
Source: PLoS One. 2015 Jun 3;10(6):e0126228. doi: 10.1371/journal.pone.0126228 (PMC4454569; doi:10.1371/journal.pone.0126228)
Supplement: S1 Table — (DOC) [file pone.0126228.s003.doc]

**S1 Table. Abundance (Number / m^2^**) of three dominant tree species from 1955 to 1985 in two communities for DBR, China.

|  | Species | 1955 | 1963 | 1967 | 1978 | 1979 | 1980 | 1982 | 1984 | 1985 |
| --- | --- | --- | --- | --- | --- | --- | --- | --- | --- | --- |
|  | *Cryptocarya chinensis* | 43.00 | 47.00 | 59.50 | 96.50 | 97.85 | 98.25 | 100.00 | 101.60 | 101.20 |
| Ⅰ | *Cryptocarya concinna* | 244.60 | 309.50 | 406.50 | 571.50 | 580.80 | 582.00 | 586.50 | 558.75 | 589.00 |
|  | *Castanopsis chinensis* | 11.35 | 9.00 | 7.00 | 2.50 | 1.00 | 0.55 | 0.53 | 0.40 | 0.40 |
|  | *Cryptocarya chinensis* | 0.20 | NA | NA | 0.50 | NA | 1.00 | 4.00 | 4.00 | 4.50 |
| Ⅱ | *Cryptocarya concinna* | 0.20 | NA | NA | 4.50 | NA | 7.00 | 8.00 | 10.00 | 12.00 |
|  | *Castanopsis chinensis* | 2.00 | 3.00 | 3.50 | 6.00 | 6.00 | 7.55 | 8.50 | 9.50 | 9.40 |

Community Ⅰ: *Cryptocarya* community;

Community Ⅱ: *Pinus-Castanopsis-Schima* community.

Missing values are indicated by NA.
