# Supplementary material for: Effects of Climate Change on Plant Population Growth Rate and Community Composition Change
Source: PLoS One. 2015 Jun 3;10(6):e0126228. doi: 10.1371/journal.pone.0126228 (PMC4454569; doi:10.1371/journal.pone.0126228)
Supplement: S2 Table — (DOC) [file pone.0126228.s004.doc]

**S2 Table. List of 77 dominant species and their abundances from the BCI (Barro Colorado Island, Panama) tropical forest.**

|  |  | **Observation time** | | | | | |
| --- | --- | --- | --- | --- | --- | --- | --- |
| **Species Name** | **Family** | **1982** | **1985** | **1990** | **1995** | **2000** | **2005** |
| *Hybanthus prunifolius* | Violaceae | 39829 | 41091 | 40438 | 36060 | 31928 | 29846 |
| *Faramea occidentalis* | Rubiaceae | 23445 | 25136 | 26894 | 27134 | 26750 | 26038 |
| *Trichilia tuberculata* | Meliaceae | 12932 | 13170 | 13294 | 12822 | 11983 | 11344 |
| *Desmopsis panamensis* | Annonaceae | 11715 | 12122 | 12145 | 11759 | 11288 | 11327 |
| *Alseis blackiana* | Rubiaceae | 7599 | 8052 | 8415 | 8177 | 7872 | 7754 |
| *Mouriri myrtilloides* | Melastomataceae | 6927 | 7682 | 7587 | 7125 | 6505 | 6540 |
| *Psychotria horizontalis* | Rubiaceae | 6161 | 6434 | 5902 | 4857 | 3937 | 3119 |
| *Hirtella triandra* | Chrysobalanaceae | 4141 | 4658 | 5025 | 5044 | 4759 | 4566 |
| *Garcinia intermedia* | Clusiaceae | 3649 | 4014 | 4289 | 4295 | 4405 | 4602 |
| *Tetragastris panamensis* | Burseraceae | 3284 | 3728 | 4116 | 4139 | 4230 | 4493 |
| *Capparis frondosa* | Capparaceae | 3534 | 3672 | 3544 | 3299 | 2989 | 2749 |
| *Sorocea affinis* | Moraceae | 3303 | 3370 | 3304 | 3115 | 2835 | 2539 |
| *Protium panamense* | Burseraceae | 2740 | 2869 | 3056 | 3009 | 2900 | 2853 |
| *Swartzia simplex*var*.ochnacea* | Fabaceae:Papil. | 2706 | 2824 | 2864 | 2876 | 2884 | 2926 |
| *Protium tenuifolium* | Burseraceae | 2601 | 2842 | 3018 | 2937 | 2851 | 2829 |
| *Tachigali versicolor* | Fabaceae:Caesal. | 2923 | 2978 | 3094 | 2947 | 2485 | 2234 |
| *Swartzia simplex*var*.grandiflora* | Fabaceae:Papil. | 2259 | 2428 | 2575 | 2605 | 2670 | 2784 |
| *Rinorea sylvatica* | Violaceae | 2577 | 2621 | 2601 | 2426 | 2315 | 2277 |
| *Beilschmiedia pendula* | Lauraceae | 2375 | 2667 | 2748 | 2589 | 2319 | 2115 |
| *Quararibea asterolepis* | Bombacaceae | 2394 | 2380 | 2347 | 2290 | 2200 | 2137 |
| *Drypetes standleyi* | Euphorbiaceae | 2177 | 2267 | 2279 | 2239 | 2193 | 2180 |
| *Eugenia oerstediana* | Myrtaceae | 2086 | 2206 | 2345 | 2217 | 1928 | 1816 |
| *Poulsenia armata* | Moraceae | 3422 | 2679 | 2120 | 1774 | 1403 | 1162 |
| *Virola sebifera* | Myristicaceae | 2400 | 2273 | 2081 | 1861 | 1613 | 1394 |
| *Coussarea curvigemmia* | Rubiaceae | 1508 | 1668 | 1971 | 2042 | 2077 | 2058 |
| *Guarea guidonia* | Meliaceae | 1782 | 1828 | 1964 | 1923 | 1834 | 1774 |
| *Oenocarpus mapora* | Arecaceae | 1780 | 1701 | 1788 | 1847 | 1830 | 1787 |
| *Pouteria reticulata* | Sapotaceae | 1651 | 1719 | 1766 | 1625 | 1400 | 1204 |
| *Pterocarpus rohrii* | Fabaceae:Papil. | 1576 | 1627 | 1703 | 1606 | 1463 | 1380 |
| *Lacistema aggregatum* | Flacourtiaceae | 1561 | 1668 | 1699 | 1632 | 1405 | 1276 |
| *Piper cordulatum* | Piperaceae | 3144 | 3707 | 1773 | 395 | 93 | 50 |
| *Cordia lasiocalyx* | Boraginaceae | 1702 | 1669 | 1694 | 1541 | 1284 | 1171 |
| *Maquira guianensis* | Moraceae | 1417 | 1443 | 1502 | 1490 | 1460 | 1396 |
| *Tabernaemontana arborea* | Apocynaceae | 1295 | 1337 | 1428 | 1506 | 1507 | 1593 |
| *Eugenia galalonensis* | Myrtaceae | 963 | 1161 | 1383 | 1503 | 1585 | 1751 |
| *Prioria copaifera* | Fabaceae:Caesal. | 1353 | 1405 | 1439 | 1417 | 1380 | 1348 |
| *Guatteria dumetorum* | Annonaceae | 1585 | 1526 | 1470 | 1256 | 1040 | 896 |
| *Simarouba amara* | Simaroubaceae | 1237 | 1248 | 1289 | 1238 | 1230 | 1477 |
| *Guarea 'fuzzy'* | Meliaceae | 1557 | 1491 | 1376 | 1227 | 1037 | 823 |
| *Ouratea lucens* | Ochnaceae | 1121 | 1238 | 1265 | 1204 | 1191 | 1227 |
| *Picramnia latifolia* | Picramniaceae | 1170 | 1177 | 1178 | 1116 | 1048 | 1059 |
| *Cupania seemannii* | Sapindaceae | 960 | 1040 | 1143 | 1174 | 1212 | 1213 |
| *Xylopia macrantha* | Annonaceae | 817 | 918 | 1045 | 1132 | 1219 | 1414 |
| *Randia armata* | Rubiaceae | 1128 | 1155 | 1143 | 1108 | 1003 | 958 |
| *Calophyllum longifolium* | Clusiaceae | 647 | 720 | 891 | 1000 | 1131 | 1427 |
| *Heisteria concinna* | Olacaceae | 883 | 959 | 984 | 973 | 953 | 927 |
| *Cassipourea elliptica* | Rhizophoraceae | 762 | 848 | 942 | 989 | 1007 | 1069 |
| *Inga umbellifera* | Fabaceae:Mimos. | 952 | 1004 | 1046 | 954 | 830 | 797 |
| *Brosimum alicastrum* | Moraceae | 862 | 894 | 922 | 909 | 898 | 892 |
| *Acalypha diversifolia* | Euphorbiaceae | 1564 | 1202 | 819 | 526 | 490 | 746 |
| *Palicourea guianensis* | Rubiaceae | 376 | 661 | 1474 | 1053 | 865 | 851 |
| *Protium costaricense* | Burseraceae | 919 | 865 | 876 | 820 | 760 | 698 |
| *Cordia bicolor* | Boraginaceae | 717 | 759 | 1044 | 967 | 765 | 658 |
| *Lonchocarpus heptaphyllus* | Fabaceae:Papil. | 844 | 848 | 893 | 837 | 736 | 659 |
| *Gustavia superba* | Lecythidaceae | 880 | 819 | 826 | 790 | 752 | 734 |
| *Hasseltia floribunda* | Flacourtiaceae | 1139 | 1012 | 859 | 686 | 542 | 484 |
| *Talisia nervosa* | Sapindaceae | 812 | 816 | 799 | 779 | 747 | 722 |
| *Eugenia coloradoensis* | Myrtaceae | 805 | 842 | 834 | 775 | 653 | 611 |
| *Unonopsis pittieri* | Annonaceae | 774 | 787 | 790 | 719 | 657 | 621 |
| *Inga nobilis* | Fabaceae:Mimos. | 780 | 785 | 776 | 723 | 664 | 615 |
| *Stylogyne turbacensis* | Myrsinaceae | 716 | 738 | 755 | 715 | 679 | 691 |
| *Ocotea whitei* | Lauraceae | 1117 | 926 | 754 | 565 | 428 | 374 |
| *Socratea exorrhiza* | Arecaceae | 812 | 737 | 684 | 673 | 622 | 540 |
| *Miconia argentea* | Melastomataceae | 530 | 677 | 900 | 799 | 600 | 518 |
| *Talisia princeps* | Sapindaceae | 620 | 638 | 672 | 643 | 632 | 664 |
| *Anaxagorea panamensis* | Annonaceae | 471 | 474 | 588 | 698 | 748 | 794 |
| *Inga marginata* | Fabaceae:Mimos. | 898 | 790 | 717 | 540 | 398 | 400 |
| *Psychotria marginata* | Rubiaceae | 577 | 686 | 690 | 629 | 553 | 581 |
| *Chrysophyllum argenteum* | Sapotaceae | 427 | 481 | 687 | 710 | 674 | 670 |
| *Croton billbergianus* | Euphorbiaceae | 621 | 621 | 1013 | 561 | 358 | 468 |
| *Cecropia insignis* | Cecropiaceae | 513 | 441 | 398 | 377 | 714 | 1144 |
| *Trichilia pallida* | Meliaceae | 560 | 572 | 588 | 566 | 516 | 478 |
| *Sloanea terniflora* | Elaeocarpaceae | 607 | 595 | 580 | 542 | 493 | 461 |
| *Herrania purpurea* | Sterculiaceae | 520 | 529 | 546 | 528 | 511 | 521 |
| *Eugenia nesiotica* | Myrtaceae | 522 | 542 | 548 | 533 | 513 | 482 |
| *Annona acuminata* | Annonaceae | 506 | 523 | 566 | 542 | 498 | 485 |
| *Garcinia madruno* | Clusiaceae | 655 | 687 | 528 | 374 | 374 | 393 |

The original database of species and their abundances were downed from the CTFS website: [http://www.ctfs.si.edu](http://www.ctfs.si.edu/).
