# Supplementary material for: Effects of Climate Change on Plant Population Growth Rate and Community Composition Change
Source: PLoS One. 2015 Jun 3;10(6):e0126228. doi: 10.1371/journal.pone.0126228 (PMC4454569; doi:10.1371/journal.pone.0126228)
Supplement: S3 Table — (DOC) [file pone.0126228.s005.doc]

**S3 Table. Comparisons of Logistic model and Gaussian model depending on the small-sample-size corrected version of Akaike information criterion (AICc).**

|  |  | **AICc** |  |  |
| --- | --- | --- | --- | --- |
| **Plot** | **Species** | **Logistic** | **Gaussian** |  |
| DBR I | 1 | 71.17609 | 70.81349 | 0.3626 |
|  | 2 | 99.86778 | 96.81192 | 3.05586 |
|  | 3 | 26.3792 | 26.44433 | -0.06513 |
| DBR II | 1 | 48.4806 | 54.09692 | -5.61632 |
|  | 2 | 53.39934 | 53.35155 | 0.04779 |
|  | 3 | # | # | #VALUE! |
| BCI | 1 | # | # | #VALUE! |
|  | 2 | 101.3358 | 95.19926 | 6.13654 |
|  | 3 | 90.85238 | 79.83648 | 11.0159 |
|  | 4 | 92.35961 | 90.98983 | 1.36978 |
|  | 5 | 85.24217 | # | #VALUE! |
|  | 6 | 95.07508 | 86.23501 | 8.84007 |
|  | 7 | # | # | #VALUE! |
|  | 8 | 126.3926 | # | #VALUE! |
|  | 9 | 106.8102 | 106.2344 | 0.5758 |
|  | 10 | 110.6874 | 109.0171 | 1.6703 |
|  | 11 | 107.0019 | 107.0697 | -0.0678 |
|  | 12 | 99.81945 | 99.28744 | 0.53201 |
|  | 13 | 96.23293 | 97.98183 | -1.7489 |
|  | 14 | 122.2783 | 112.4132 | 9.8651 |
|  | 15 | 100.761 | 100.7436 | 0.0174 |
|  | 16 | # | # | #VALUE! |
|  | 17 | 99.47767 | 98.43071 | 1.04696 |
|  | 18 | 92.71242 | 88.76661 | 3.94581 |
|  | 19 | 99.29351 | 97.10951 | 2.184 |
|  | 20 | 104.4209 | 100.958 | 3.4629 |
|  | 21 | 81.81241 | 79.30713 | 2.50528 |
|  | 22 | 97.89176 | 53.35155 | 44.54021 |
|  | 23 | # | # | #VALUE! |
|  | 24 | 103.2828 | 96.9846 | 6.2982 |
|  | 25 | 122.0677 | 112.8802 | 9.1875 |
|  | 26 | 96.49135 | 96.81459 | -0.32324 |
|  | 27 | 129.6124 | 129.1378 | 0.4746 |
|  | 28 | # | # | #VALUE! |
|  | 29 | 92.10962 | 90.6924 | 1.41722 |
|  | 30 | 109.7259 | 106.1387 | 3.5872 |
|  | 31 | 95.86586 | 99.35681 | -3.49095 |
|  | 32 | 99.51326 | 97.2106 | 2.30266 |
|  | 33 | # | # | #VALUE! |
|  | 34 | 101.0763 | 97.40245 | 3.67385 |
|  | 35 | 103.7905 | 103.7294 | 0.0611 |
|  | 36 | 86.11982 | 93.2051 | -7.08528 |
|  | 37 | 101.656 | 101.3582 | 0.2978 |
|  | 38 | # | 108.7571 | #VALUE! |
|  | 39 | 83.05975 | 91.46179 | -8.40204 |
|  | 40 | # | # | #VALUE! |
|  | 41 | 105.1156 | 103.7832 | 1.3324 |
|  | 42 | 104.3666 | 94.47022 | 9.89638 |
|  | 43 | 107.1168 | 109.333 | -2.2162 |
|  | 44 | 98.71419 | 99.09517 | -0.38098 |
|  | 45 | 105.0968 | 87.20853 | 17.88827 |
|  | 46 | 110.7837 | 108.629 | 2.1547 |
|  | 47 | 129.837 | 115.473 | 14.364 |
|  | 48 | 114.1364 | 105.998 | 8.1384 |
|  | 49 | 111.5728 | 105.5053 | 6.0675 |
|  | 50 | 112.4178 | 102.297 | 10.1208 |
|  | 51 | 108.5755 | 107.6721 | 0.9034 |
|  | 52 | 114.2397 | 104.1807 | 10.059 |
|  | 53 | 104.6651 | 105.6783 | -1.0132 |
|  | 54 | 87.9683 | 88.37822 | -0.40992 |
|  | 55 | # | # | #VALUE! |
|  | 56 | 119.984 | 113.4561 | 6.5279 |
|  | 57 | 107.4621 | 103.6243 | 3.8378 |
|  | 58 | 90.88615 | 88.87566 | 2.01049 |
|  | 59 | # | 115.3362 | #VALUE! |
|  | 60 | 110.4219 | 109.8083 | 0.6136 |
|  | 61 | 106.931 | 108.758 | -1.827 |
|  | 62 | 120.052 | 113.7966 | 6.2554 |
|  | 63 | 114.5727 | 114.7982 | -0.2255 |
|  | 64 | 98.21808 | 104.0265 | -5.80842 |
|  | 65 | 115.8244 | 109.5783 | 6.2461 |
|  | 66 | 109.3587 | 99.60998 | 9.74872 |
|  | 67 | 126.2603 | 112.3278 | 13.9325 |
|  | 68 | 118.4106 | 121.8885 | -3.4779 |
|  | 69 | 116.1576 | 118.9075 | -2.7499 |
|  | 70 | 126.2603 | 119.3216 | 6.9387 |
|  | 71 | 127.2133 | 123.9309 | 3.2824 |
|  | 72 | 133.6324 | 131.9066 | 1.7258 |
|  | 73 | # | 123.8173 | #VALUE! |
|  | 74 | # | 127.3518 | #VALUE! |
|  | 75 | 127.9671 | 120.7453 | 7.2218 |
|  | 76 | 136.7617 | 130.1728 | 6.5889 |
|  | 77 | 149.0235 | 147.005 | 2.0185 |

Green: Gaussian model fitted better

Pink: Logistic model fitted better

Missing values were due to error reports, which might were caused by improper parameter selection.
