# Supplementary material for: Effects of Climate Change on Plant Population Growth Rate and Community Composition Change
Source: PLoS One. 2015 Jun 3;10(6):e0126228. doi: 10.1371/journal.pone.0126228 (PMC4454569; doi:10.1371/journal.pone.0126228)
Supplement: S4 Table — (DOC) [file pone.0126228.s006.doc]

**S4 Table.** **Significance, Adjusted R-squared (Adj-R^2^), and the small-sample-size corrected version of Akaike information criterion (AICc) of 6 Models for 3 species from 2 plots in DBR.**

|  |  |  |  |  | **plot1** |  |  |  |  |  |  |  |  |  | **plot2** |  |  |  |  |  |
| --- | --- | --- | --- | --- | --- | --- | --- | --- | --- | --- | --- | --- | --- | --- | --- | --- | --- | --- | --- | --- |
|  |  | 1 |  |  | 2 |  |  | 3 |  |  |  | 1 |  |  | 2 |  |  | 3 |  |  |
|  | model | Adj-R2 |  | AICc | Adj-R2 |  | AICc | Adj-R2 |  | AICc |  | Adj-R2 |  | AICc | Adj-R2 |  | AICc | Adj-R2 |  | AICc |
| 1 | T+(t1+t2) | 0.4484 | *** | -208.728 | 0.5425 | *** | -203.228 | 0.1426 | * | -15.1638 |  | 0.5038 | ** | -59.2833 | 0.02947 |  | 2.568347 | 0.006091 |  | -128.396 |
| 2 | P+(t1+t2) | 0.7226 | *** | -233.471 | 0.764 | *** | -227.057 | 0.06625 |  | -12.0925 |  | -0.1378 |  | -46.8342 | 0.02972 |  | 2.564461 | 0.05422 |  | -130.183 |
| 3 | T+T*(t1+t2) | 0.466 | *** | -208.295 | 0.7296 | *** | -220.557 | 0.1604 | * | -14.3176 |  | 0.537 | ** | -56.9589 | -0.0453 |  | 7.043214 | -0.02329 |  | -125.745 |
| 4 | P+P*(t1+t2) | 0.8458 | *** | -253.005 | 0.7774 | *** | -227.553 | 0.07122 |  | -10.683 |  | -0.1816 |  | -42.9068 | -0.05495 |  | 7.180921 | 0.08073 |  | -129.605 |
| 5 | T+P+(t1+t2) | 0.7153 | *** | -230.934 | 0.781 | *** | -228.14 | 0.1583 | * | -14.2264 |  | 0.5855 | ** | -58.6207 | -0.05767 |  | 7.219647 | 0.05645 |  | -128.666 |
| 6 | T+P+(T+P)*(t1+t2) | 0.8482 | *** | -249.898 | 0.8462 | *** | -237.197 | 0.3283 | ** | -18.6743 |  | 0.6112 | * | -49.2577 | -0.2521 |  | 20.07362 | 0.134 | . | -128.077 |

Darker yellow background indicates better model fitting.

.P<0.1;*P<0.05:**P<0.01:***P<0.001.
