# Supplementary material for: Effects of Climate Change on Plant Population Growth Rate and Community Composition Change
Source: PLoS One. 2015 Jun 3;10(6):e0126228. doi: 10.1371/journal.pone.0126228 (PMC4454569; doi:10.1371/journal.pone.0126228)
Supplement: S5 Table — (DOC) [file pone.0126228.s007.doc]

**S5 Table.** **Significance, Adjusted R-squared (Adj-R^2^), and the small-sample-size corrected version of Akaike information criterion (AICc) of 6 Models for 77 species in BCI.**

|  |  | 1 |  |  | 2 |  |  | 3 |  |  | 4 |  |  | 5 |  |  | 6 |  |  | 7 |  |  |
| --- | --- | --- | --- | --- | --- | --- | --- | --- | --- | --- | --- | --- | --- | --- | --- | --- | --- | --- | --- | --- | --- | --- |
|  | model | Adj-R2 |  | AICc | Adj-R2 |  | AICc | Adj-R2 |  | AICc | Adj-R2 |  | AICc | Adj-R2 |  | AICc | Adj-R2 |  | AICc | Adj-R2 |  | AICc |
| 1 | T+(t1+t2) | 0.9912 | *** | -109.322 | 0.9827 | *** | -155.359 | 0.4795 | ** | -94.2156 | 0.7565 | *** | -108.364 | 0.975 | *** | -127.2 | 0.9878 | *** | -177.163 | 0.5036 | ** | -109.986 |
| 2 | P+(t1+t2) | 0.751 | *** | -59.2617 | 0.6978 | *** | -112.416 | 0.4982 | ** | -94.7648 | 0.8328 | *** | -114.004 | 0.8018 | *** | -96.1337 | 0.7022 | *** | -129.297 | 0.2169 | . | -103.147 |
| 3 | T+T*(t1+t2) | 0.9953 | *** | -115.577 | 0.9909 | *** | -161.584 | 0.653 | ** | -96.9351 | 0.872 | *** | -114.649 | 0.9728 | *** | -122.576 | 0.9886 | *** | -174.881 | 0.7501 | *** | -116.921 |
| 4 | P+P*(t1+t2) | 0.8596 | *** | -64.4868 | 0.8575 | *** | -120.336 | 0.7761 | *** | -103.507 | 0.9064 | *** | -119.342 | 0.9449 | *** | -111.969 | 0.8211 | *** | -133.577 | 0.5756 | ** | -108.973 |
| 5 | T+P+(t1+t2) | 0.9983 | *** | -130.434 | 0.9937 | *** | -167.153 | 0.9385 | *** | -122.902 | 0.9922 | *** | -156.691 | 0.9761 | *** | -124.528 | 0.9924 | *** | -180.949 | 0.9805 | *** | -155.174 |
| 6 | T+P+(T+P)*(t1+t2) | 0.9981 | *** | -118.861 | 0.9967 | *** | -166.34 | 0.9468 | *** | -114.752 | 0.9919 | *** | -145.704 | 0.9944 | *** | -135.911 | 0.9908 | *** | -167.822 | 0.9814 | *** | -145.551 |
|  |  | 8 |  |  | 9 |  |  | 10 |  |  | 11 |  |  | 12 |  |  | 13 |  |  | 14 |  |  |
|  | model | Adj-R2 |  | AICc | Adj-R2 |  | AICc | Adj-R2 |  | AICc | Adj-R2 |  | AICc | Adj-R2 |  | AICc | Adj-R2 |  | AICc | Adj-R2 |  | AICc |
| 1 | T+(t1+t2) | 0.9486 | *** | -136.388 | 0.9576 | *** | -139.778 | 0.8395 | *** | -63.1353 | 0.6978 | *** | -77.8434 | 0.674 | *** | -68.6523 | 0.6089 | ** | -98.9792 | 0.9288 | *** | -120.668 |
| 2 | P+(t1+t2) | 0.7487 | *** | -112.586 | 0.6612 | *** | -108.598 | 0.8746 | *** | -66.8364 | 0.9455 | *** | -103.547 | 0.966 | *** | -102.563 | 0.8766 | *** | -116.286 | 0.9385 | *** | -122.876 |
| 3 | T+T*(t1+t2) | 0.9441 | *** | -131.782 | 0.9694 | *** | -141.314 | 0.8975 | *** | -66.5014 | 0.7908 | *** | -80 | 0.7927 | *** | -72.084 | 0.8217 | *** | -107.402 | 0.9362 | *** | -118.95 |
| 4 | P+P*(t1+t2) | 0.9425 | *** | -131.347 | 0.9097 | *** | -125.066 | 0.893 | *** | -65.8556 | 0.9424 | *** | -99.3437 | 0.9644 | *** | -98.4929 | 0.9518 | *** | -127.01 | 0.9369 | *** | -119.132 |
| 5 | T+P+(t1+t2) | 0.958 | *** | -136.053 | 0.9591 | *** | -136.961 | 0.8664 | *** | -62.5282 | 0.9763 | *** | -112.693 | 0.9829 | *** | -109.501 | 0.9539 | *** | -127.686 | 0.9793 | *** | -135.854 |
| 6 | T+P+(T+P)*(t1+t2) | 0.991 | *** | -148.816 | 0.9928 | *** | -152.679 | 0.9417 | *** | -64.6374 | 0.9889 | *** | -113.757 | 0.9879 | *** | -104.377 | 0.9657 | *** | -121.781 | 0.991 | *** | -138.072 |
|  |  | 15 |  |  | 16 |  |  | 17 |  |  | 18 |  |  | 19 |  |  | 20 |  |  | 21 |  |  |
|  | model | Adj-R2 |  | AICc | Adj-R2 |  | AICc | Adj-R2 |  | AICc | Adj-R2 |  | AICc | Adj-R2 |  | AICc | Adj-R2 |  | AICc | Adj-R2 |  | AICc |
| 1 | T+(t1+t2) | 0.4075 | * | -41.2795 | 0.9737 | *** | -150.508 | 0.9345 | *** | -148.762 | 0.9352 | *** | -151.439 | 0.8418 | *** | -108.429 | 0.9751 | *** | -132.842 | 0.9849 | *** | -162.616 |
| 2 | P+(t1+t2) | 0.4042 | * | -41.198 | 0.8921 | *** | -129.345 | 0.3006 | * | -113.242 | 0.5467 | ** | -122.256 | 0.6509 | *** | -96.5588 | 0.6645 | *** | -93.8378 | 0.9354 | *** | -140.797 |
| 3 | T+T*(t1+t2) | 0.6153 | ** | -44.3973 | 0.9741 | *** | -147.396 | 0.9457 | *** | -148.211 | 0.9321 | *** | -147.365 | 0.9058 | *** | -112.853 | 0.9753 | *** | -129.592 | 0.9892 | *** | -164.208 |
| 4 | P+P*(t1+t2) | 0.5494 | ** | -42.0267 | 0.8983 | *** | -126.872 | 0.7976 | *** | -128.477 | 0.8576 | *** | -136.258 | 0.9449 | *** | -120.88 | 0.892 | *** | -107.485 | 0.9605 | *** | -144.814 |
| 5 | T+P+(t1+t2) | 0.9644 | *** | -80.101 | 0.9713 | *** | -145.842 | 0.9308 | *** | -144.578 | 0.9571 | *** | -154.271 | 0.8718 | *** | -154.271 | 0.9729 | *** | -128.215 | 0.9926 | *** | -169.984 |
| 6 | T+P+(T+P)*(t1+t2) | 0.9767 | *** | -76.1044 | 0.9795 | *** | -140.562 | 0.9891 | *** | -161.969 | 0.9886 | *** | -163.777 | 0.9633 | *** | -116.646 | 0.9945 | *** | -141.731 | 0.9915 | *** | -157.447 |
|  |  | 22 |  |  | 23 |  |  | 24 |  |  | 25 |  |  | 26 |  |  | 27 |  |  | 28 |  |  |
|  | model | Adj-R2 |  | AICc | Adj-R2 |  | AICc | Adj-R2 |  | AICc | Adj-R2 |  | AICc | Adj-R2 |  | AICc | Adj-R2 |  | AICc | Adj-R2 |  | AICc |
| 1 | T+(t1+t2) | 0.8692 | *** | -108.211 | 0.996 | *** | -175.544 | 0.9691 | *** | -146.708 | 0.7446 | *** | -79.3229 | 0.9959 | *** | -171.296 | 0.7869 | *** | -118.523 | 0.7782 | *** | -111.272 |
| 2 | P+(t1+t2) | 0.8168 | *** | -103.158 | 0.9279 | *** | -132.052 | 0.435 | * | -103.133 | 0.2286 | . | -62.7431 | 0.9475 | *** | -133.178 | 0.9357 | *** | -136.489 | 0.8634 | *** | -118.537 |
| 3 | T+T*(t1+t2) | 0.9463 | *** | -118.217 | 0.9966 | *** | -174.374 | 0.9722 | *** | -144.922 | 0.8559 | *** | -84.5459 | 0.9957 | *** | -167.521 | 0.859 | *** | -121.365 | 0.8689 | *** | -115.798 |
| 4 | P+P*(t1+t2) | 0.9497 | *** | -119.188 | 0.9639 | *** | -139.063 | 0.7043 | *** | -109.48 | 0.4643 | * | -64.8519 | 0.9784 | *** | -143.126 | 0.9413 | *** | -134.512 | 0.9057 | *** | -120.732 |
| 5 | T+P+(t1+t2) | 0.9679 | *** | -125.928 | 0.997 | *** | -176.586 | 0.9811 | *** | -150.717 | 0.9295 | *** | -95.2759 | 0.998 | *** | -178.943 | 0.9879 | *** | -158.158 | 0.9932 | *** | -160.249 |
| 6 | T+P+(T+P)*(t1+t2) | 0.9879 | *** | -130.233 | 0.9972 | *** | -166.962 | 0.9805 | *** | -139.935 | 0.9222 | *** | -83.4705 | 0.9994 | *** | -187.115 | 0.9931 | *** | -156.397 | 0.9937 | *** | -150.94 |
|  |  | 29 |  |  | 30 |  |  | 31 |  |  | 32 |  |  | 33 |  |  | 34 |  |  | 35 |  |  |
|  | model | Adj-R2 |  | AICc | Adj-R2 |  | AICc | Adj-R2 |  | AICc | Adj-R2 |  | AICc | Adj-R2 |  | AICc | Adj-R2 |  | AICc | Adj-R2 |  | AICc |
| 1 | T+(t1+t2) | 0.07328 |  | -107.002 | 0.4374 | * | -110.884 | 0.9321 | *** | -131.319 | 0.723 | *** | -130.627 | 0.975 | *** | -132.162 | 0.9678 | *** | -139.057 | 0.3687 | * | -80.0768 |
| 2 | P+(t1+t2) | 0.7929 | *** | -129.478 | 0.2762 |  | -107.104 | 0.7767 | *** | -113.464 | 0.3246 | * | -117.256 | 0.8533 | *** | -105.626 | 0.5402 | ** | -99.1838 | 0.3406 | * | -79.425 |
| 3 | T+T*(t1+t2) | 0.2975 |  | -107.795 | 0.7628 | *** | -120.475 | 0.9399 | *** | -129.783 | 0.8606 | *** | -137.566 | 0.9753 | *** | -129.013 | 0.9855 | *** | -147.67 | 0.7155 | *** | -88.6713 |
| 4 | P+P*(t1+t2) | 0.9497 | *** | -147.349 | 0.7278 | *** | -118.414 | 0.9235 | *** | -126.174 | 0.6364 | ** | -123.181 | 0.969 | *** | -125.597 | 0.7954 | *** | -107.972 | 0.7124 | *** | -88.5104 |
| 5 | T+P+(t1+t2) | 0.8578 | *** | -131.754 | 0.9154 | *** | -135.938 | 0.975 | *** | -142.954 | 0.9912 | *** | -179.066 | 0.9728 | *** | -127.529 | 0.9893 | *** | -152.239 | 0.9399 | *** | -111.99 |
| 6 | T+P+(T+P)*(t1+t2) | 0.941 | *** | -134.627 | 0.951 | *** | -133.826 | 0.9879 | *** | -143.552 | 0.9922 | *** | -170.55 | 0.9945 | *** | -141.146 | 0.9969 | *** | -160.264 | 0.9562 | *** | -106.419 |
|  |  | 36 |  |  | 37 |  |  | 38 |  |  | 39 |  |  | 40 |  |  | 41 |  |  | 42 |  |  |
|  | model | Adj-R2 |  | AICc | Adj-R2 |  | AICc | Adj-R2 |  | AICc | Adj-R2 |  | AICc | Adj-R2 |  | AICc | Adj-R2 |  | AICc | Adj-R2 |  | AICc |
| 1 | T+(t1+t2) | 0.9638 | *** | -151.687 | 0.8825 | *** | -111.237 | 0.919 | *** | -114.639 | 0.8056 | *** | -107.708 | 0.921 | *** | -145.398 | 0.9315 | *** | -83.6629 | 0.8908 | *** | -112.571 |
| 2 | P+(t1+t2) | 0.8492 | *** | -130.269 | 0.6293 | ** | -93.9961 | 0.8518 | *** | -105.575 | 0.9796 | *** | -141.503 | 0.9704 | *** | -160.12 | 0.8435 | *** | -71.2585 | 0.3412 | * | -85.6185 |
| 3 | T+T*(t1+t2) | 0.9798 | *** | -157.086 | 0.953 | *** | -121.612 | 0.9446 | *** | -116.98 | 0.8864 | *** | -112.401 | 0.9232 | *** | -142.464 | 0.9697 | *** | -92.5479 | 0.8819 | *** | -108.037 |
| 4 | P+P*(t1+t2) | 0.9166 | *** | -135.804 | 0.8644 | *** | -105.717 | 0.9793 | *** | -131.763 | 0.9819 | *** | -139.996 | 0.9813 | *** | -163.624 | 0.9341 | *** | -80.8767 | 0.8508 | *** | -104.538 |
| 5 | T+P+(t1+t2) | 0.9982 | *** | -192.991 | 0.9767 | *** | -132.161 | 0.9256 | *** | -112.555 | 0.9929 | *** | -154.026 | 0.9677 | *** | -155.462 | 0.9939 | *** | -116.581 | 0.8952 | *** | -109.827 |
| 6 | T+P+(T+P)*(t1+t2) | 0.9979 | *** | -181.016 | 0.9935 | *** | -141.055 | 0.9789 | *** | -121.102 | 0.9928 | *** | -143.507 | 0.9903 | *** | -163.147 | 0.9977 | *** | -120.881 | 0.9817 | *** | -125.679 |
|  |  | 43 |  |  | 44 |  |  | 45 |  |  | 46 |  |  | 47 |  |  | 48 |  |  | 49 |  |  |
|  | model | Adj-R2 |  | AICc | Adj-R2 |  | AICc | Adj-R2 |  | AICc | Adj-R2 |  | AICc | Adj-R2 |  | AICc | Adj-R2 |  | AICc | Adj-R2 |  | AICc |
| 1 | T+(t1+t2) | 0.4022 | * | -99.3475 | 0.5124 | ** | -112.927 | 0.9386 | *** | -128.294 | 0.9016 | *** | -58.5851 | 0.7223 | *** | -128.686 | 0.9602 | *** | -68.3302 | 0.7936 | *** | -107.041 |
| 2 | P+(t1+t2) | 0.6984 | *** | -109.611 | 0.3461 | * | -108.523 | 0.2736 | . | -91.2372 | 0.6618 | *** | -40.0718 | 0.1497 |  | -111.898 | 0.1937 |  | -23.1923 | 0.6446 | *** | -98.8915 |
| 3 | T+T*(t1+t2) | 0.6804 | ** | -105.38 | 0.634 | ** | -113.867 | 0.9445 | *** | -126.442 | 0.9161 | *** | -57.6141 | 0.8907 | *** | -139.311 | 0.9675 | *** | -68.0164 | 0.8796 | *** | -111.77 |
| 4 | P+P*(t1+t2) | 0.8111 | *** | -113.265 | 0.7694 | *** | -120.794 | 0.6642 | ** | -99.4473 | 0.7342 | *** | -40.3253 | 0.6846 | ** | -123.414 | 0.7044 | *** | -34.8857 | 0.7655 | *** | -101.765 |
| 5 | T+P+(t1+t2) | 0.9666 | *** | -139.246 | 0.9182 | *** | -136.347 | 0.9714 | *** | -136.38 | 0.9745 | *** | -75.4924 | 0.944 | *** | -149.353 | 0.9586 | *** | -64.3751 | 0.9541 | *** | -126.247 |
| 6 | T+P+(T+P)*(t1+t2) | 0.9637 | *** | -127.698 | 0.9541 | *** | -134.687 | 0.9792 | *** | -130.882 | 0.9836 | *** | -71.7399 | 0.9817 | *** | -155.775 | 0.9931 | *** | -80.8726 | 0.9469 | *** | -113.733 |
|  |  | 50 |  |  | 51 |  |  | 52 |  |  | 53 |  |  | 54 |  |  | 55 |  |  | 56 |  |  |
|  | model | Adj-R2 |  | AICc | Adj-R2 |  | AICc | Adj-R2 |  | AICc | Adj-R2 |  | AICc | Adj-R2 |  | AICc | Adj-R2 |  | AICc | Adj-R2 |  | AICc |
| 1 | T+(t1+t2) | 0.9683 | *** | -127.953 | 0.9929 | *** | -180.669 | 0.185 |  | -107.144 | 0.9199 | *** | -133.966 | 0.9914 | *** | -157.849 | 0.9825 | *** | -132.174 | 0.9035 | *** | -100.215 |
| 2 | P+(t1+t2) | 0.957 | *** | -123.363 | 0.8246 | *** | -132.544 | 0.7456 | *** | -124.609 | 0.8409 | *** | -123.664 | 0.6629 | *** | -102.906 | 0.832 | *** | -98.2876 | 0.2641 | . | -69.7412 |
| 3 | T+T*(t1+t2) | 0.9849 | *** | -135.756 | 0.9939 | *** | -179.452 | 0.3106 | . | -106.293 | 0.9573 | *** | -140.014 | 0.9915 | *** | -154.788 | 0.9844 | *** | -130.615 | 0.8989 | *** | -96.1581 |
| 4 | P+P*(t1+t2) | 0.9821 | *** | -133.194 | 0.9243 | *** | -141.787 | 0.9554 | *** | -147.366 | 0.9122 | *** | -129.221 | 0.839 | *** | -110.634 | 0.9181 | *** | -105.712 | 0.818 | *** | -87.332 |
| 5 | T+P+(t1+t2) | 0.9978 | *** | -164.556 | 0.9931 | *** | -177.704 | 0.811 | *** | -125.706 | 0.998 | *** | -186.251 | 0.9909 | *** | -153.747 | 0.9903 | *** | -137.703 | 0.8999 | *** | -96.3034 |
| 6 | T+P+(T+P)*(t1+t2) | 0.9989 | *** | -164.472 | 0.9965 | *** | -177.458 | 0.9485 | *** | -134.889 | 0.998 | *** | -175.331 | 0.9936 | *** | -148.602 | 0.991 | *** | -128.5 | 0.9833 | *** | -112.834 |
|  |  | 57 |  |  | 58 |  |  | 59 |  |  | 60 |  |  | 61 |  |  | 62 |  |  | 63 |  |  |
|  | model | Adj-R2 |  | AICc | Adj-R2 |  | AICc | Adj-R2 |  | AICc | Adj-R2 |  | AICc | Adj-R2 |  | AICc | Adj-R2 |  | AICc | Adj-R2 |  | AICc |
| 1 | T+(t1+t2) | 0.886 | *** | -120.706 | 0.827 | *** | -166.005 | 0.812 | *** | -123.271 | 0.9712 | *** | -161.41 | 0.8018 | *** | -108.966 | 0.7456 | *** | -134.304 | 0.9647 | *** | -146.182 |
| 2 | P+(t1+t2) | 0.8487 | *** | -116.466 | 0.8465 | *** | -167.806 | 0.7075 | *** | -116.647 | 0.197 |  | -111.478 | 0.2852 | . | -89.7227 | 0.8627 | *** | -143.55 | -0.1217 |  | -94.3181 |
| 3 | T+T*(t1+t2) | 0.9416 | *** | -127.388 | 0.9218 | *** | -174.551 | 0.8693 | *** | -125.37 | 0.9748 | *** | -160.054 | 0.9225 | *** | -119.686 | 0.8848 | *** | -142.827 | 0.9662 | *** | -143.481 |
| 4 | P+P*(t1+t2) | 0.9206 | *** | -122.769 | 0.9686 | *** | -188.235 | 0.9526 | *** | -140.593 | 0.5156 | * | -115.7 | 0.7089 | *** | -99.8331 | 0.9485 | *** | -154.911 | 0.4071 | * | -100.519 |
| 5 | T+P+(t1+t2) | 0.9968 | *** | -170.877 | 0.9195 | *** | -174.122 | 0.8241 | *** | -120.915 | 0.9852 | *** | -168.03 | 0.9641 | *** | -131.23 | 0.9745 | *** | -165.449 | 0.9757 | *** | -148.416 |
| 6 | T+P+(T+P)*(t1+t2) | 0.9971 | *** | -162.176 | 0.9677 | *** | -177.501 | 0.9454 | *** | -128.125 | 0.9822 |  | -154.914 | 0.9858 | *** | -134.861 | 0.9827 | *** | -160.94 | 0.9751 | *** | -137.753 |
|  |  | 64 |  |  | 65 |  |  | 66 |  |  | 67 |  |  | 68 |  |  | 69 |  |  | 70 |  |  |
|  | model | Adj-R2 |  | AICc | Adj-R2 |  | AICc | Adj-R2 |  | AICc | Adj-R2 |  | AICc | Adj-R2 |  | AICc | Adj-R2 |  | AICc | Adj-R2 |  | AICc |
| 1 | T+(t1+t2) | 0.9925 | *** | -167.174 | 0.8801 | *** | -132.679 | 0.98 | *** | -163.709 | 0.8584 | *** | -147.82 | 0.2273 | . | -123.319 | 0.805 | *** | -103.513 | 0.8933 | *** | -162.213 |
| 2 | P+(t1+t2) | 0.9625 | *** | -143.021 | 0.2193 | . | -104.571 | 0.4387 | * | -113.725 | 0.4857 | *** | -128.476 | 0.609 | ** | -133.539 | 0.9281 | *** | -118.478 | 0.7688 | *** | -150.623 |
| 3 | T+T*(t1+t2) | 0.992 | *** | -162.896 | 0.9506 | *** | -142.617 | 0.9795 | *** | -159.989 | 0.8597 | *** | -144.606 | 0.6404 | ** | -131.433 | 0.9154 | *** | -112.688 | 0.8857 | *** | -157.826 |
| 4 | P+P*(t1+t2) | 0.9915 | *** | -161.989 | 0.6435 | ** | -112.967 | 0.7184 | *** | -120.711 | 0.8631 | *** | -144.97 | 0.8203 | *** | -141.837 | 0.9744 | *** | -130.604 | 0.9744 | *** | -180.286 |
| 5 | T+P+(t1+t2) | 0.9921 | *** | -162.964 | 0.9848 | *** | -160.257 | 0.9837 | *** | -163.427 | 0.9295 | *** | -154.928 | 0.634 | ** | -131.167 | 0.9459 | *** | -119.397 | 0.893 | *** | -158.88 |
| 6 | T+P+(T+P)*(t1+t2) | 0.9975 | *** | -169.915 | 0.9959 | *** | -169.8 | 0.9853 | *** | -154.675 | 0.9819 | *** | -165.024 | 0.8123 | *** | -130.862 | 0.9707 | *** | -118.25 | 0.9761 | *** | -170.989 |
|  |  | 71 |  |  | 72 |  |  | 73 |  |  | 74 |  |  | 75 |  |  | 76 |  |  | 77 |  |  |
|  | model | Adj-R2 |  | AICc | Adj-R2 |  | AICc | Adj-R2 |  | AICc | Adj-R2 |  | AICc | Adj-R2 |  | AICc | Adj-R2 |  | AICc | Adj-R2 |  | AICc |
| 1 | T+(t1+t2) | 0.756 | *** | -123.93 | 0.9857 | *** | -148.658 | 0.9123 | *** | -128.386 | 0.9494 | *** | -147.178 | 0.9227 | *** | -133.443 | 0.9267 | *** | -153.388 | 0.9177 | *** | -137.721 |
| 2 | P+(t1+t2) | 0.1071 |  | -104.473 | 0.4696 | ** | -94.4796 | 0.9573 | *** | -139.187 | 0.9067 | *** | -138.01 | 0.6016 | ** | -108.839 | 0.9895 | *** | -182.514 | 0.1137 |  | -102.074 |
| 3 | T+T*(t1+t2) | 0.8432 | *** | -127.202 | 0.9844 | *** | -143.992 | 0.9612 | *** | -137.249 | 0.979 | *** | -156.983 | 0.9499 | *** | -136.57 | 0.9653 | *** | -161.252 | 0.9461 | *** | -140.721 |
| 4 | P+P*(t1+t2) | 0.4164 | * | -107.491 | 0.7463 | *** | -102.178 | 0.9872 | *** | -153.907 | 0.9731 | *** | -153.281 | 0.7506 | *** | -112.5 | 0.9887 | *** | -178.081 | 0.7258 | *** | -116.308 |
| 5 | T+P+(t1+t2) | 0.9841 | *** | -161.558 | 0.9854 | *** | -144.988 | 0.9888 | *** | -155.864 | 0.9879 | *** | -165.226 | 0.9938 | *** | -168.033 | 0.9902 | *** | -180.174 | 0.925 | *** | -135.755 |
| 6 | T+P+(T+P)*(t1+t2) | 0.9857 | *** | -152.778 | 0.9894 | *** | -139.539 | 0.9938 | *** | -154.383 | 0.9963 | *** | -172.563 | 0.9932 | *** | -156.262 | 0.9923 | *** | -173.5 | 0.9884 | *** | -153.486 |

Darker yellow background indicates better model fitting.

.P<0.1;*P<0.05:**P<0.01:***P<0.001.
