# Supplementary material for: Effects of Climate Change on Plant Population Growth Rate and Community Composition Change
Source: PLoS One. 2015 Jun 3;10(6):e0126228. doi: 10.1371/journal.pone.0126228 (PMC4454569; doi:10.1371/journal.pone.0126228)
Supplement: S6 Table — (DOC) [file pone.0126228.s008.doc]

**S6 Table. Significance of coefficients, Adjusted R-squared (Adj-R^2^), and the Akaike information criterion (AIC) of 6 Models fitted with all species from each of the 3 plots (DBRⅠ, DBRⅡ and BCI), separately.**

|  |  |  | DBRⅠ |  |  |  |  | DBRⅡ |  |  |  |  | BCI |  |
| --- | --- | --- | --- | --- | --- | --- | --- | --- | --- | --- | --- | --- | --- | --- |
|  | **model** |  | Adj-R^2^ |  | AIC |  |  | Adj-R^2^ |  | AIC |  |  | Adj-R^2^ |  |
| 1 | T+(t1+t2) |  | 0.03289 | . | -102.6904 |  |  | 0.04749 | . | -90.48676 |  |  | 0.0588 | *** |
|  | Intercept | * |  |  |  |  | * |  |  |  |  | *** |  |  |
|  | T |  |  |  |  |  |  |  |  |  |  | *** |  |  |
|  | t1+t2 | * |  |  |  |  | * |  |  |  |  | *** |  |  |
| 2 | P+(t1+t2) |  | 0.02919 | . | -102.2775 |  |  | 0.06362 | * | -91.61385 |  |  | 0.03744 | *** |
|  | Intercept | . |  |  |  |  | * |  |  |  |  | *** |  |  |
|  | P |  |  |  |  |  |  |  |  |  |  | * |  |  |
|  | t1+t2 | . |  |  |  |  | * |  |  |  |  | *** |  |  |
| 3 | T+T*(t1+t2) |  | 0.02751 |  | -101.1243 |  |  | 0.03228 |  | -88.49718 |  |  | 0.06133 | *** |
|  | Intercept | * |  |  |  |  | * |  |  |  |  | * |  |  |
|  | T |  |  |  |  |  |  |  |  |  |  | * |  |  |
|  | t1+t2 | * |  |  |  |  | * |  |  |  |  | * |  |  |
|  | T:(t1+t2) |  |  |  |  |  |  |  |  |  |  | * |  |  |
| 4 | P+P*(t1+t2) |  | 0.02478 |  | -100.8212 |  |  | 0.04872 |  | -89.62814 |  |  | 0.05103 | *** |
|  | Intercept |  |  |  |  |  | * |  |  |  |  | *** |  |  |
|  | P |  |  |  |  |  |  |  |  |  |  | *** |  |  |
|  | t1+t2 |  |  |  |  |  | * |  |  |  |  | *** |  |  |
|  | P:(t1+t2) |  |  |  |  |  |  |  |  |  |  | *** |  |  |
| 5 | T+P+(t1+t2) |  | 0.03938 | . | -102.451 |  |  | 0.05601 | . | -90.13574 |  |  | 0.06376 | *** |
|  | Intercept | * |  |  |  |  | * |  |  |  |  | *** |  |  |
|  | T |  |  |  |  |  |  |  |  |  |  | *** |  |  |
|  | P | * |  |  |  |  | * |  |  |  |  | ** |  |  |
|  | t1+t2 |  |  |  |  |  |  |  |  |  |  | *** |  |  |
| 6 | T+P+(T+P)*(t1+t2) | | 0.0533 | . | -102.1236 |  |  | 0.03121 |  | -86.58798 |  |  | 0.06224 | *** |
|  | Intercept | . |  |  |  |  | * |  |  |  |  |  |  |  |
|  | T |  |  |  |  |  |  |  |  |  |  |  |  |  |
|  | P |  |  |  |  |  |  |  |  |  |  |  |  |  |
|  | t1+t2 | . |  |  |  |  | * |  |  |  |  |  |  |  |
|  | T:(t1+t2) |  |  |  |  |  |  |  |  |  |  |  |  |  |
|  | P:(t1+t2) |  |  |  |  |  |  |  |  |  |  |  |  |  |

Darker yellow background indicates better model fitting.

.P<0.1;*P<0.05:**P<0.01:***P<0.001.
